# A new species of alpine grasshopper of the genus *Podismopsis* Zubovski, 1900 (Orthoptera, Acrididae, Gomphocerinae) from Central Europe

**DOI:** 10.3897/zookeys.1295.205424

**Published:** 2026-10-26

**Authors:** Petr Kočárek, Pavel Marhoul, Robert Vlk, Jaroslav Holuša

**Affiliations:** 1 University of Ostrava, Faculty of Science, Department of Biology and Ecology, Chittussiho 10, 710 00 Ostrava, Czech Republic University of Ostrava, Faculty of Science, Department of Biology and Ecology Ostrava Czech Republic; 2 Beleco NGO, Na Zátorce 10, 160 00 Prague, Czech Republic Beleco NGO Prague Czech Republic; 3 Regional Museum in Mikulov, Zámek 1/4, 692 01 Mikulov, Czech Republic Regional Museum in Mikulov Mikulov Czech Republic; 4 Czech University of Life Sciences Prague, Faculty of Forestry and Wood Sciences, Kamýcká 129, 165 00 Prague, Czech Republic Czech University of Life Sciences Prague, Faculty of Forestry and Wood Sciences Prague Czech Republic

**Keywords:** Identification key, interglacial diversification, Pleistocene speciation, semi-species, sky islands, taxonomy, Slovakia, Western Palaearctic

## Abstract

*Podismopsis kristini***sp. nov**. (Orthoptera, Acrididae, Gomphocerinae) is described from the Tatras in Slovakia, representing the seventh European species of the genus *Podismopsis* Zubovski, 1900, and the first record of the genus from the Western Carpathians. The new species inhabits cold, wind-exposed alpine grasslands at 1,820–1,920 m a.s.l. in the upper part of the Žiar Valley in the Western Tatras and is known only from the type locality. It is diagnosed and distinguished from the other six European congeners (*P. frontalis*, *P. keisti*, *P. poppiusi*, *P. relicta*, *P. styriaca*, and *P. transsylvanica*) by a combination of morphometric and qualitative characters. A DNA barcode based on a partial cytochrome c oxidase I (COI) sequence is also provided for molecular identification purposes. The European *Podismopsis* species exemplify the “sky island” model of allopatric speciation: the genus colonised Central Europe via ice-free Pleistocene steppes and, following postglacial climatic warming, retreated upslope into isolated mountain refugia where reproductive isolation caused by brachyptery subsequently drove species divergence. Given their strict allopatry, low molecular divergence and undifferentiated calling songs, the European species collectively represent semi-species sensu Lorković (1955) forming a superspecies, i.e., lineages in which speciation is still incomplete. We provide an updated identification key to all European *Podismopsis* species and discuss the biogeographic implications for understanding Quaternary diversification patterns in flightless alpine insects.

## Introduction

The genus *Podismopsis* Zubovski, 1900 (Acrididae: Gomphocerinae: Chrysochraontini) is distributed from Europe to northeastern Asia via Russia, comprising almost 40 species (Lu et al. 2011; Cigliano et al. 2026). The centre of diversity and likely origin of the genus lies in the taiga zone of Siberia, the Russian Far East, and adjacent regions, where most species inhabit extensive swampy forests with rich hygrophilous vegetation (Bey-Bienko 1932). From this Angaran core area, cold-adapted lineages extended westward across the Palaearctic, reaching central and southern Europe (Bey-Bienko 1932; Nadig 1989). In Europe, the genus is represented by six species, four of which are confined to a single isolated mountain massif or region, collectively occurring across six countries (Nadig 1989; Koschuh 2008; Gottsberger and Berger 2011).

The inconspicuous appearance of *Podismopsis*, particularly of females, may also contribute to the apparently late discovery of some European populations. Females are strongly brachypterous and may superficially resemble other short-winged grasshoppers occurring in montane and alpine habitats. This similarity is particularly evident in *Podismopsis keisti* (Nadig, 1989), which is morphologically similar to *Chrysochraon dispar* (Germar, 1834), especially in females, and the two species are explicitly compared in identification keys (Nadig 1989; Coray and Thorens 2001). Other short-winged grasshoppers occurring syntopically with European *Podismopsis*, such as *Miramella* Dovnar-Zapolskij, 1932 and *Pseudochorthippus* Defaut, 2012, may further contribute to difficulties in field recognition (Koschuh 2008). Such morphological similarity and the inconspicuous occurrence of these species may have contributed to the historical under-recording of *Podismopsis* in some mountain regions.

The earliest described European species is *Podismopsis poppiusi* (Miram, 1907), originally recorded from polar and subpolar regions of the European part of Russia, including the Arkhangelsk region, and distributed eastward across northern Siberia (Miram 1907; Bey-Bienko 1932; Cigliano et al. 2026). Unlike the other European congeners, *P. poppiusi* is not a strictly montane endemic but rather a boreal species associated with the northern taiga and tundra belt. Willy Ramme described two further species: *P. relicta* Ramme, 1931 from Montenegro (Ramme 1931), and *P. transsylvanica* Ramme, 1951 from Romania (Ramme 1951; Nadig 1989). Later, Lev Mistshenko described *P. frontalis* Mistshenko, 1951 endemic to the southern Urals (Bey-Bienko and Mistshenko 1951). More recently described Alpine species include *P. keisti* from Switzerland (Nadig 1989; Baur et al. 2006) and *P. styriaca* Koschuh, 2008 from Styria (Steiermark), Austria (Koschuh 2008; Pucher 2014).

The four alpine European species – *P. relicta*, *P. transsylvanica*, *P. styriaca*, and *P. keisti* – share a conspicuous set of ecological and biogeographical characteristics: all are strictly endemic to small, isolated mountain summits above 1,800 m a.s.l., and all inhabit cold, wind-exposed alpine grasslands (Nadig 1989; Koschuh 2008; Hochkirch et al. 2016a, 2016b; Breitenmoser 2017). The absence of functional wings renders these grasshoppers entirely dependent on walking for dispersal, effectively preventing colonisation of neighbouring mountain ranges and confining each population to its respective summit – a pattern classically described as the “sky island” phenomenon (Taylor 1942; Gottsberger and Berger 2011). It has long been hypothesised that *Podismopsis* entered Europe during the Pleistocene via the ice-free steppe corridor extending from Siberia to Central Europe, and that postglacial warming forced cold-adapted populations to retreat upslope into mountain refugia, where reproductive isolation subsequently drove speciation (Holdhaus 1912; Ander 1949; Nadig 1989). Although molecular data generated by Gottsberger and Berger (2011) revealed unexpectedly low COI divergence among the three studied European species (*P. keisti*, *P. styriaca*, and *P. relicta*), suggesting that wide gene flow was maintained across the glacial steppes until postglacial disjunction, the populations are now entirely isolated and continue to diverge in isolation – a process consistent with recent interglacial diversification models proposed for other flightless alpine insects (Taberlet et al. 1998; Garnier et al. 2004).

The present paper describes a new species of *Podismopsis* from the Western Carpathians, representing the seventh European species of the genus and a further testament to the cryptic diversity harboured by the isolated mountain summits of Central Europe.

## Materials and methods

We collected *Podismopsis* grasshoppers in alpine vegetation habitats in the Western Tatras. Sampling was conducted by slowly walking through the study site and visually searching for individuals. Males were also located by their characteristic calling songs. Once detected, grasshoppers were captured using an entomological sweep net. The surveyors progressed across the locality in a line to ensure systematic coverage of the area. Specimens were transferred to the laboratory for identification. Examined specimens were preserved partly dried and partly in 95% ethanol. The specimens used for visualisation were photographed with a Leica Z16 APO macroscope (Leica Microsystems, Wetzlar, Germany) equipped with a Canon 6D Mark II camera. Stacks of 10 to 20 focal planes of the same specimen were combined with Helicon Focus software and subsequently processed with Adobe Photoshop CS6 Extended (v. 13). For the study of male genitalia, abdomens were dissected and placed into 10% NaOH solution for 8–12 h. Cleared genitalia were examined under a digital microscope (Keyence VHX-X1). Measurements were taken from images captured with a Keyence VHX-5000 digital microscope (VH-Z20T and VH-ZST objectives) using the associated software at the Department of Botany and Zoology, Masaryk University in Brno.

For sound recordings, the mini-disc recorder Sony MZ-R909 with a handmade microphone (frequency response up to 20 kHz) was used. Songs were recorded in laboratory conditions at a temperature of 20–21 °C. For the analysis, songs of three isolated males were used. After digitising the songs on a PC, oscillogram analysis was performed with the software Raven Lite 2.0 Standard (Cornell Lab of Ornithology, NY, USA). For the song description, we used the following terminology: pulse – the sound produced by one stroke of a hind leg (the shortest measurable unit, i.e., the first-order unit); syllable – the sound produced by one complete up and down movement of the hind legs, starting when the legs leave their initial position and ending when the legs return to their initial position, and representing the repeated unit of a consistent structure (the second-order unit); echeme – a series of consistent syllables separated by pauses (the third-order unit).

Distribution maps were prepared using QGIS v. 3.40, an open-source geographic information system (QGIS Development Team 2025), and displayed in the WGS 84 / Pseudo-Mercator projection (EPSG:3857). The European basemap was derived from the Copernicus GLO-90 Digital Elevation Model provided by the European Space Agency (ESA 2021), whereas detailed inset maps were based on OpenStreetMap data (OpenStreetMap contributors 2026). The distribution ranges of all species except *Podismopsis kristini* sp. nov. were compiled from species assessments published in the IUCN Red List of Threatened Species, namely *P. frontalis* (Chobanov et al. 2016c), *P. keisti* (Zuna-Kratky et al. 2016a), *P. poppiusi* (Hochkirch et al. 2016b), *P. relicta* (Chobanov et al. 2016a), *P. styriaca* (Zuna-Kratky et al. 2016b), and *P. transsylvanica* (Chobanov et al. 2016b). The published distribution maps were georeferenced and subsequently manually digitised as vector polygons (ESRI Shapefile format) in QGIS. Distances between localities were calculated as geodesic distances using geographic coordinates referenced to WGS 84. The distribution range of *P. kristini* sp. nov. was reconstructed from field records obtained during the present study.

Genomic DNA was extracted from middle leg tissue using the QIAamp DNA Micro Kit (Qiagen Inc.) following the manufacturer’s protocol. A fragment of the mitochondrial cytochrome *c* oxidase subunit I (COI) gene, the standard animal DNA barcode marker, was amplified using the universal primer pair LCO1490 and HCO2198 (Folmer et al. 1994). PCR amplification and purification of PCR products were performed as described by Kočárek et al. (2026). Purified amplicons were Sanger sequenced by Eurofins Genomics (Konstanz, Germany). Chromatograms were visually inspected and edited using ChromasPro v. 2.1.10.1 (Technelysium, Brisbane, Australia).

Sequences were aligned in MEGA v. 12 (Kumar et al. 2024) using MUSCLE with default settings. Uncorrected pairwise *p*-distances were calculated using the complete-deletion option from the 543-bp COI alignment. The best-fitting nucleotide-substitution model was selected using the Bayesian information criterion (BIC) implemented in MEGA v. 12. A maximum-likelihood phylogenetic tree was inferred under the Tamura 3-parameter model with a proportion of invariant sites (T92+I). Branch support was assessed using 1,000 nonparametric bootstrap replicates. A TCS haplotype network was constructed from eight COI sequences using PopART v. 1.7 (Leigh and Bryant 2015). The tree was rooted using *Chrysochraon dispar* as the outgroup.

The holotype and some paratypes (3♂♂, 2♀♀) are deposited in the National Museum, Prague, Czech Republic (**NMPC**). The remaining paratypes (8♂♂, 8♀♀) are preserved in the personal collections of the contributors: Petr Kočárek (**PKCO**), Pavel Marhoul (**PMCO**), Jaroslav Holuša (**JHCO**), and Robert Vlk (**RVCO**). The remaining studied material is deposited at the following collections: Naturmuseum St. Gallen, Switzerland (**NMSG**), and the private collection of David Musiolek, Karviná, Czech Republic (**DMCO**).

### Comparative material of other *Podismopsis* species studied

*Podismopsis transsylvanica* Ramme, 1951, Paratypes • 1♂/1♀, Rumänien, Fagaras, Surul, 2,150 m, 8.9.42, Ramme leg. et det., Paratypus [red label] (NMPC); • 1♀, Fogaras, Szurul, 22.8.1957, leg. et det. B. Kis (coll. NMPC); • 2♂♂, Fogaras, Szurul, 26.8.1960, leg. et det. B. Kis (NMPC); *Podismopsis relicta* Ramme, 1931, Topotype • 1♂/1♀, Yugoslavia (Kosmet), Hajla Pl., 1,800–2,100 m, 16.-IX-1964, F. Willemse leg. et det., Topotype [red label] (NMPC); *Podismopsis keisti* (Nadig, 1989) • 1♂, CH SG Grabs, Trosboden, 7439/2259 1,840 m, 07.08.1991, leg. B. Keist, det. B. Keist (NMSG); • 1♀, CH SG Grabs, Trosboden, 7437/2243 1,900 m, 14.10.1992, leg. B. Keist, det. B. Keist 1992 (NMSG); *Podismopsis styriaca* Koschuh, 2008 • 1♂/1♀, Austria, Hüttenberg, Hohenwart, Klippitzsee, 1,800 m, 15.09.2019, leg. R. Vlk, det. R. Vlk (RVCO); • 1♂/1♀, Austria, Hüttenberg, Hohenwart, Klippitzsee, 1800 m, 15.09.2019, leg. D. Musiolek, det. D. Musiolek (DMCO).

## Results

### Taxonomy

#### Genus Podismopsis

Taxon classificationAnimaliaOrthopteraAcrididae

Zubovski, 1900

627A29CA-A806-5E35-8689-B2220DD5FB83

Chrysochraon (Podismopsis) Zubovski, 1900: 2. Type species: Chrysochraon (Podismopsis) altaica Zubovski, 1900, by original monotypy. Type locality: Altai, Russia.

##### Included European species.

*P. frontalis* Mistshenko, 1951; *P. keisti* (Nadig, 1989); *P. kristini* sp. nov.; *P. poppiusi* (Miram, 1907); *P. relicta* Ramme, 1931; *P. styriaca* Koschuh, 2008; *P. transsylvanica* Ramme, 1951.

##### Distribution.

Palaearctic (Central and southeastern Europe to northeastern Asia).

#### Podismopsis kristini sp. nov.

Taxon classificationAnimaliaOrthopteraAcrididae

8095411B-EF96-56F6-AEDF-8254380A8AED

https://zoobank.org/BB8BC434-9C7A-466C-8820-E19812980844

Figs 1, 2, 3, 4, 5, 6, 8–10

##### Material examined.

***Holotype***: • ♂, **Slovakia**: Western Tatras Mts., Žiar Valley, 49.1931278°N, 19.7475797°E, 1,850 m a.s.l., 09.IX.2022, R. Vlk, P. Marhoul, J. Holuša and P. Kočárek leg.; Holotype, *Podismopsis kristini* sp. nov., R. Vlk, P. Marhoul, J. Holuša and P. Kočárek det. 2026 [red label] (coll. NMPC). ***Paratypes***: • 11♂♂/10♀♀, **Slovakia**: same data as in holotype; Paratype, *Podismopsis kristini* sp. nov., R. Vlk, P. Marhoul, J. Holuša and P. Kočárek det. 2026 [red label] (3♂♂, 2♀♀ coll. NMPC, 1♂, 1♀ coll. PKCO, 3♂♂, 3♀♀ coll. PMCO, 1♂, 1♀ coll. JHCO, 3♂♂, 3♀♀ coll. RVCO).

**Figure 1.**
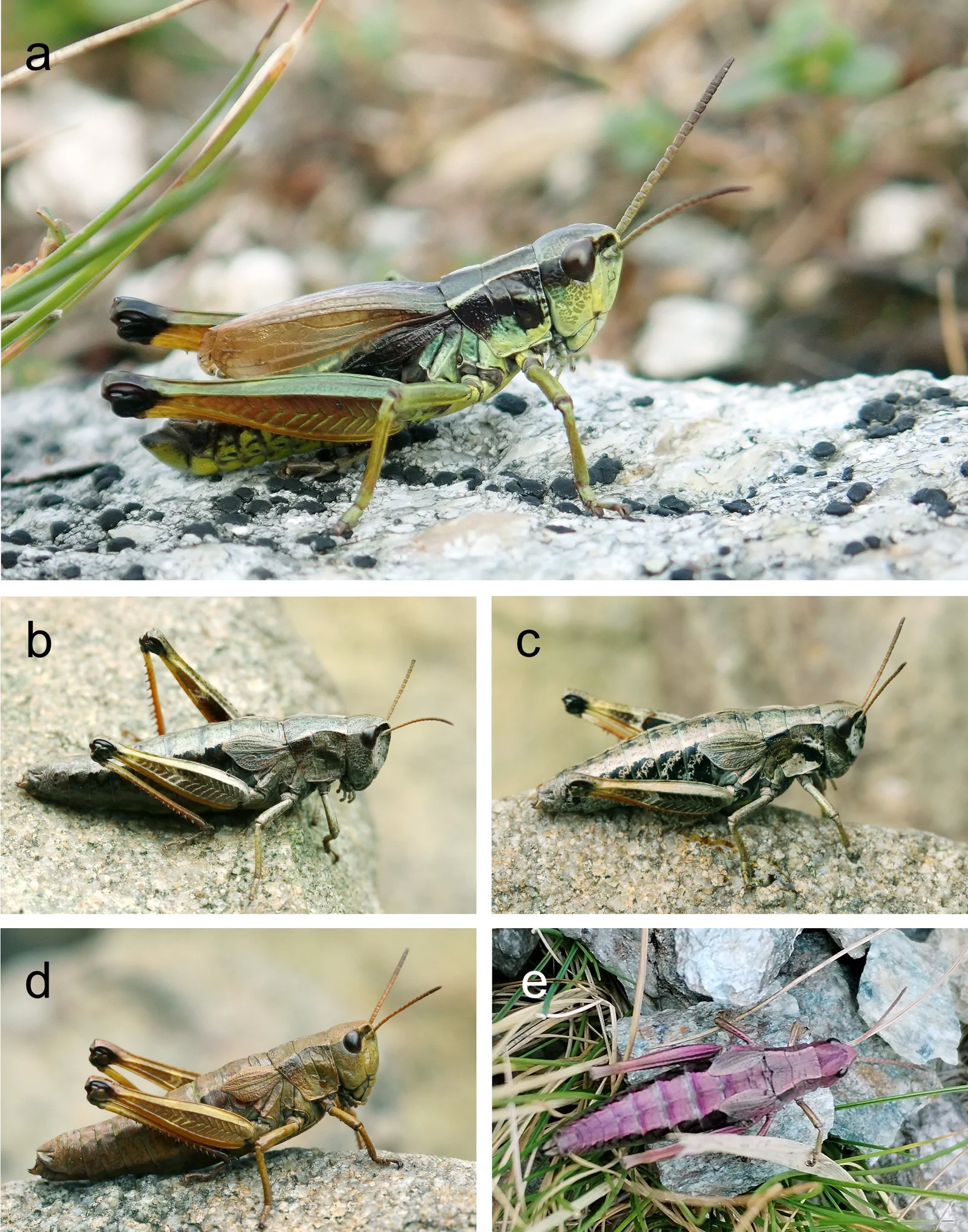
Living specimens of *Podismopsis kristini* sp. nov. **a**. Male; **b–e**. Females (e – erythristic specimen). Photos: P. Kočárek (**a–d**) and A. Krištín (**e**).

**Figure 2.**
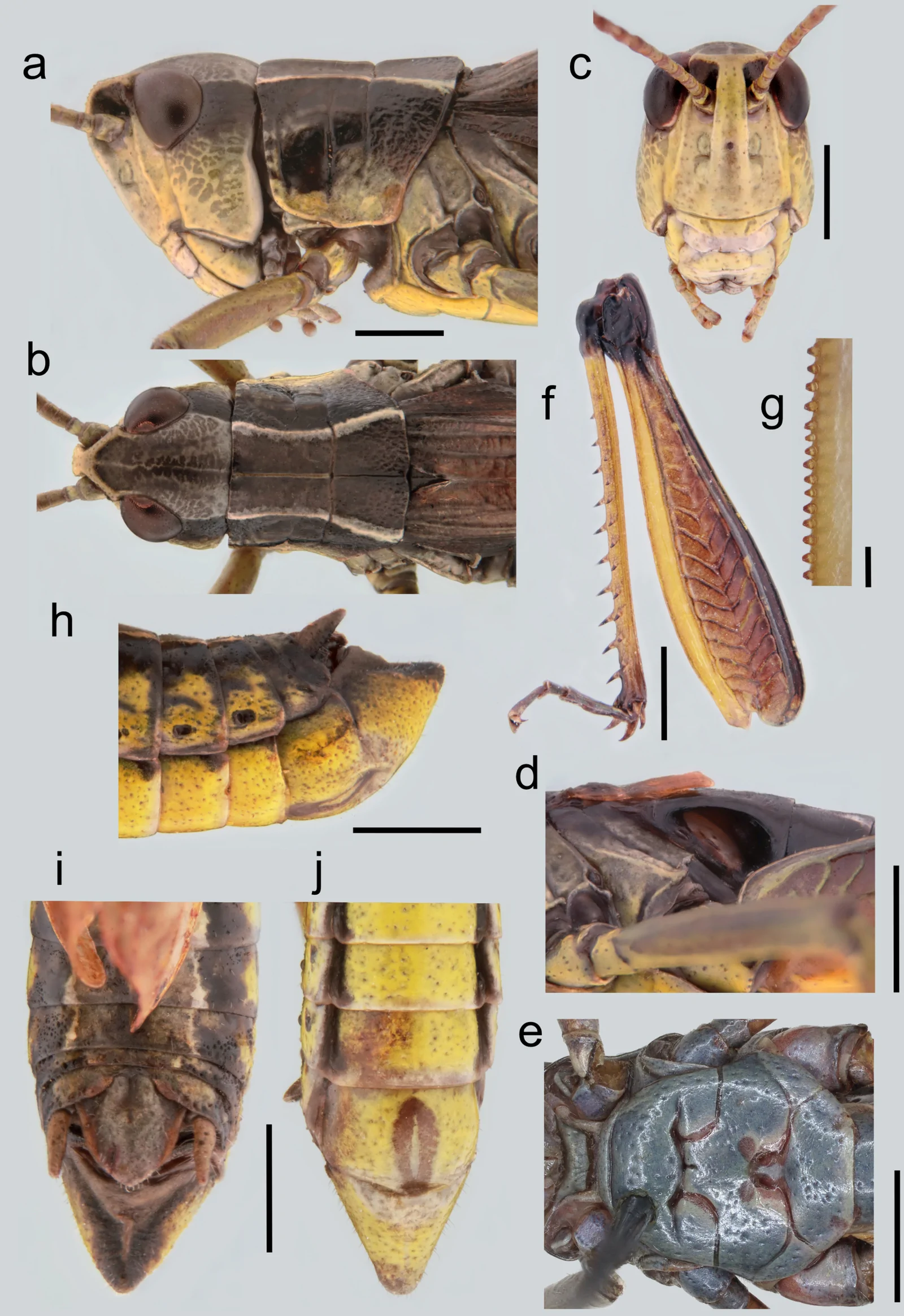
*Podismopsis kristini* sp. nov., male. **a**. Head and pronotum, lateral view; **b**. Head and pronotum, dorsal view; **c**. Head from the front; **d**. Opening of tympanum, lateral view, tegmen removed; **e**. Sternal segments, ventral view; **f**. Right hind leg, external view; **g**. Central part of stridulatory file, lateral view; **h**. End of abdomen, lateral view; **i**. End of abdomen, dorsal view; **j**. End of abdomen, ventral view. Scale bars: 2.0 mm (**a–f, h–j**); 0.1 mm (**g**).

**Figure 3.**
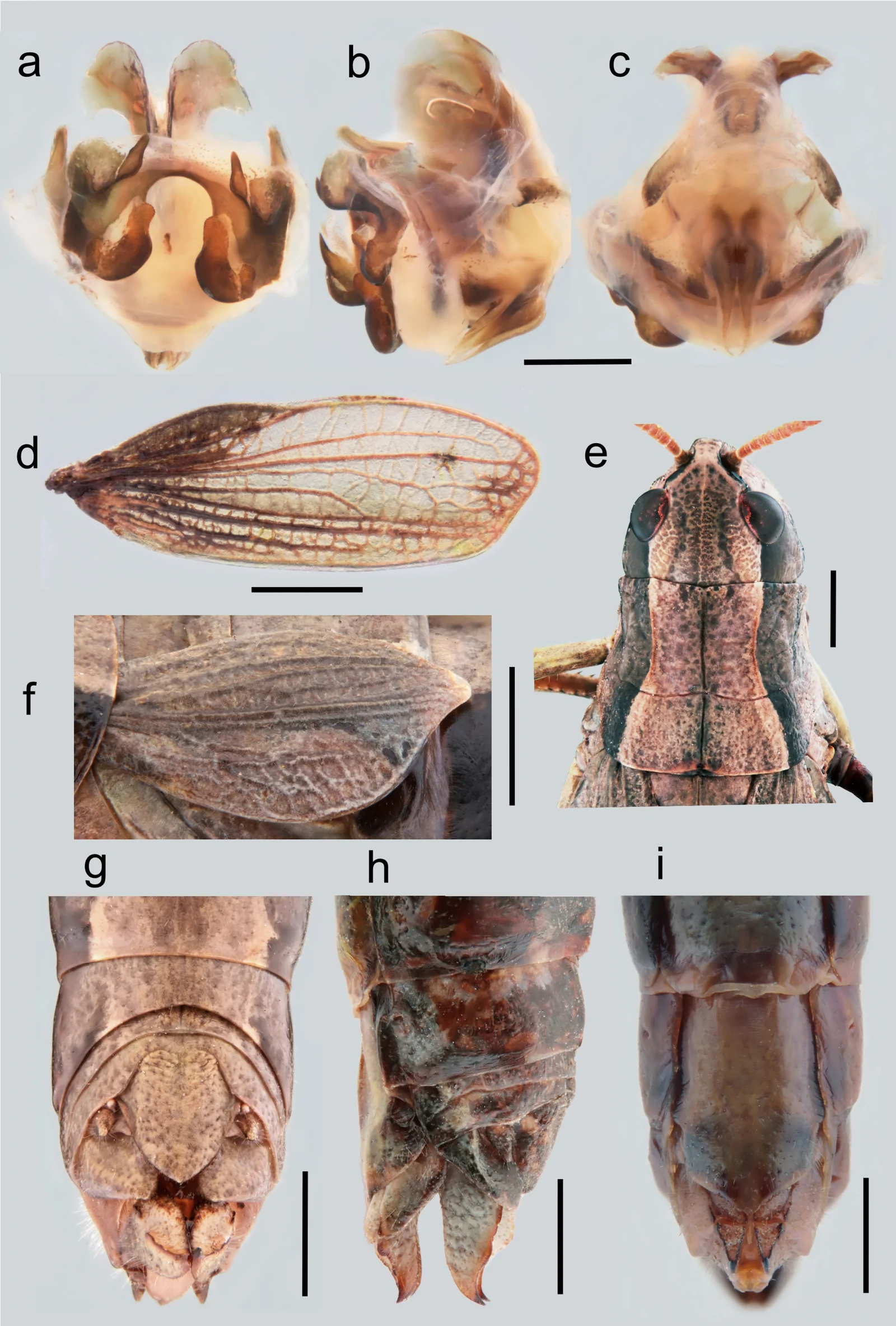
*Podismopsis kristini* sp. nov., male (**a–d**), female (**e–i**). **a**. Phallic complex, dorsal view; **b**. Phallic complex, lateral view; **c**. Phallic complex, ventral view; **d**. Male right tegmen; **e**. Female head and pronotum in dorsal view; **f**. Female left tegmen, lateral view; **g**. End of abdomen, dorsal view; **h**. End of abdomen, lateral view; **i**. End of abdomen, ventral view. Scale bars: 1.0 mm (**a–c**); 2.0 mm (**d–i**).

**Figure 4.**
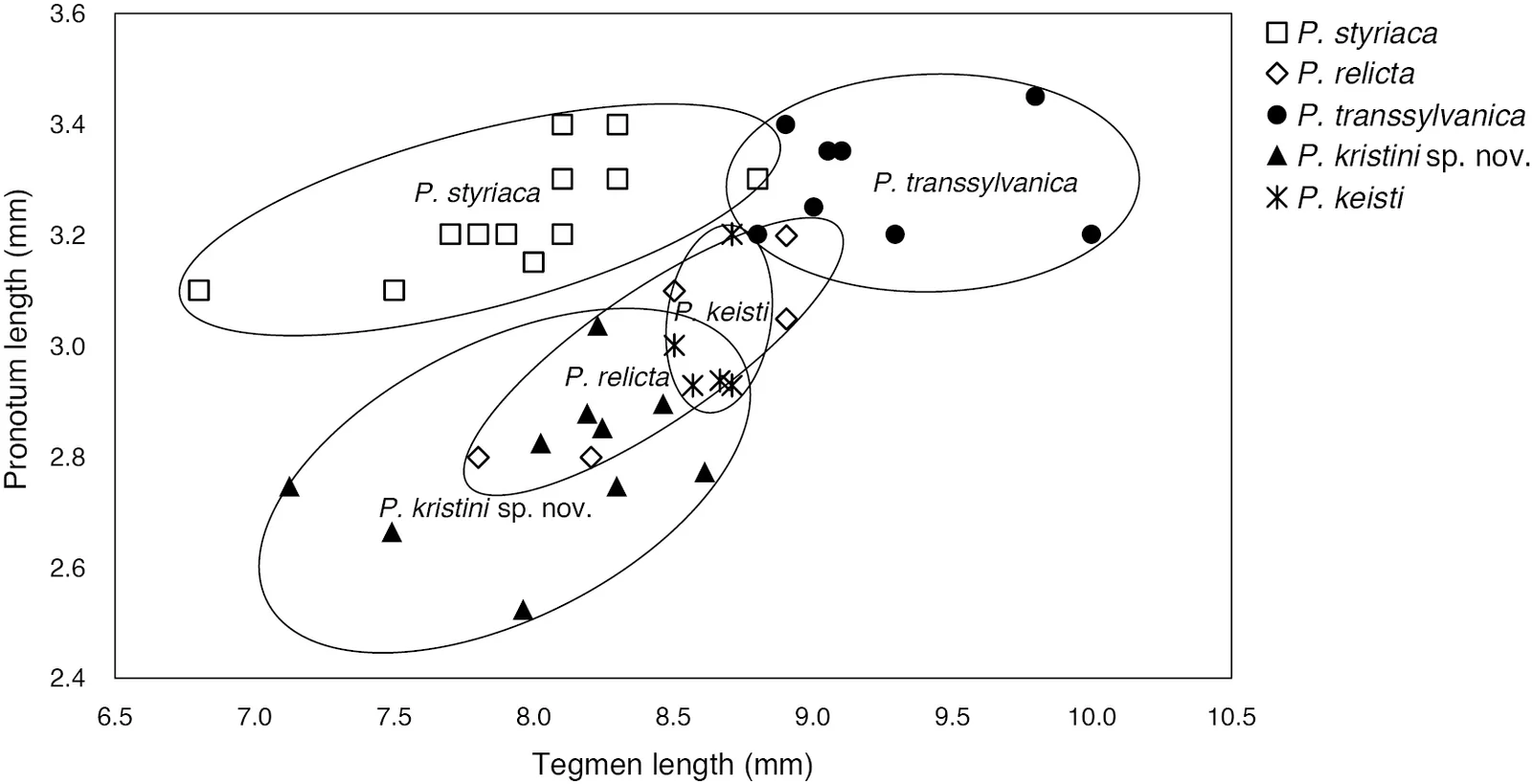
Relationship between tegmen length and pronotum length in five species, combining original measurements with data extracted from Koschuh (2008). Each symbol represents an individual specimen. Ellipses outline species clusters for visual guidance only and have no statistical meaning.

**Figure 5.**
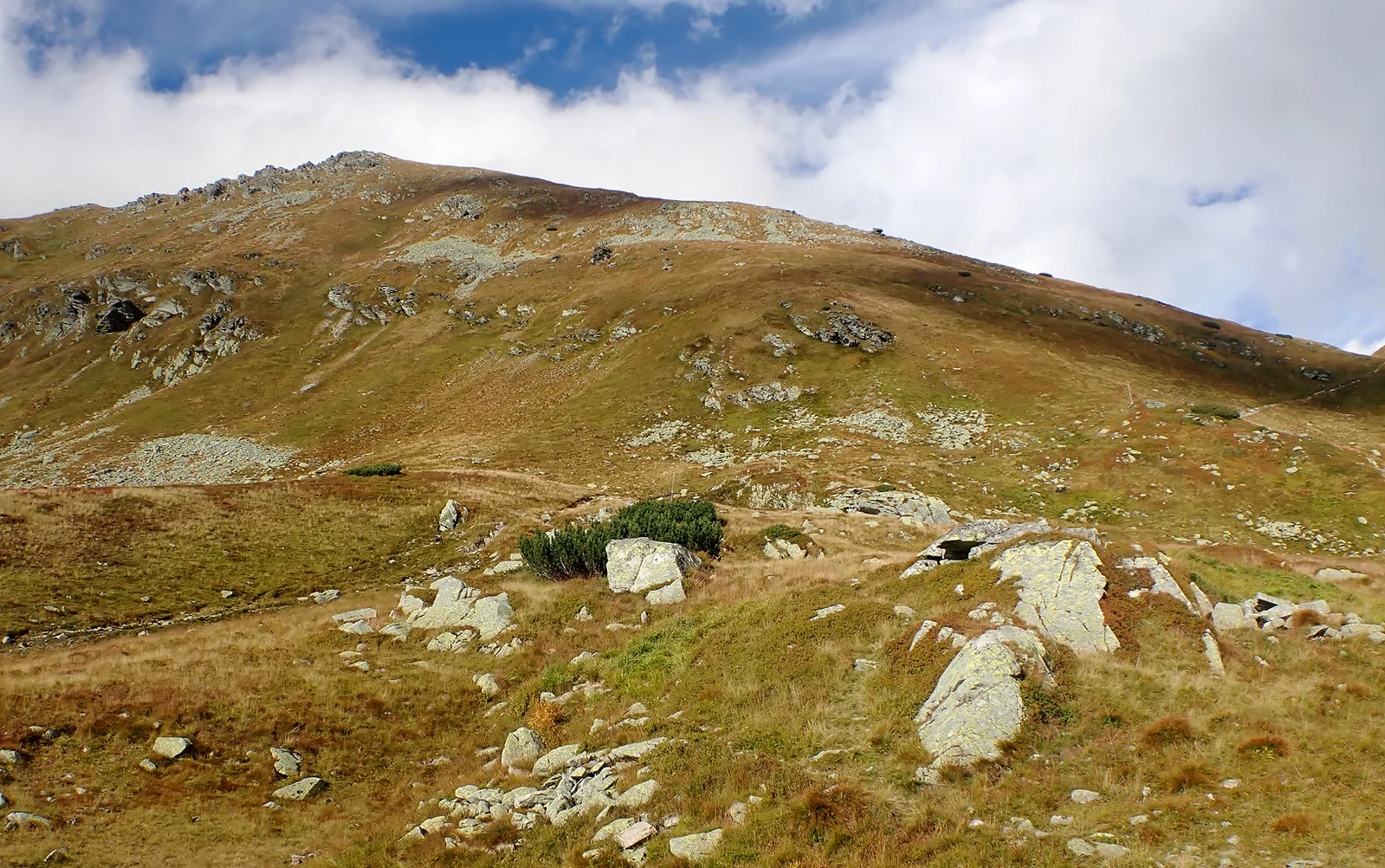
Habitat of *Podismopsis kristini* sp. nov. in the upper part of Žiar Valley (the Western Tatras, Slovakia).

**Figure 6.**
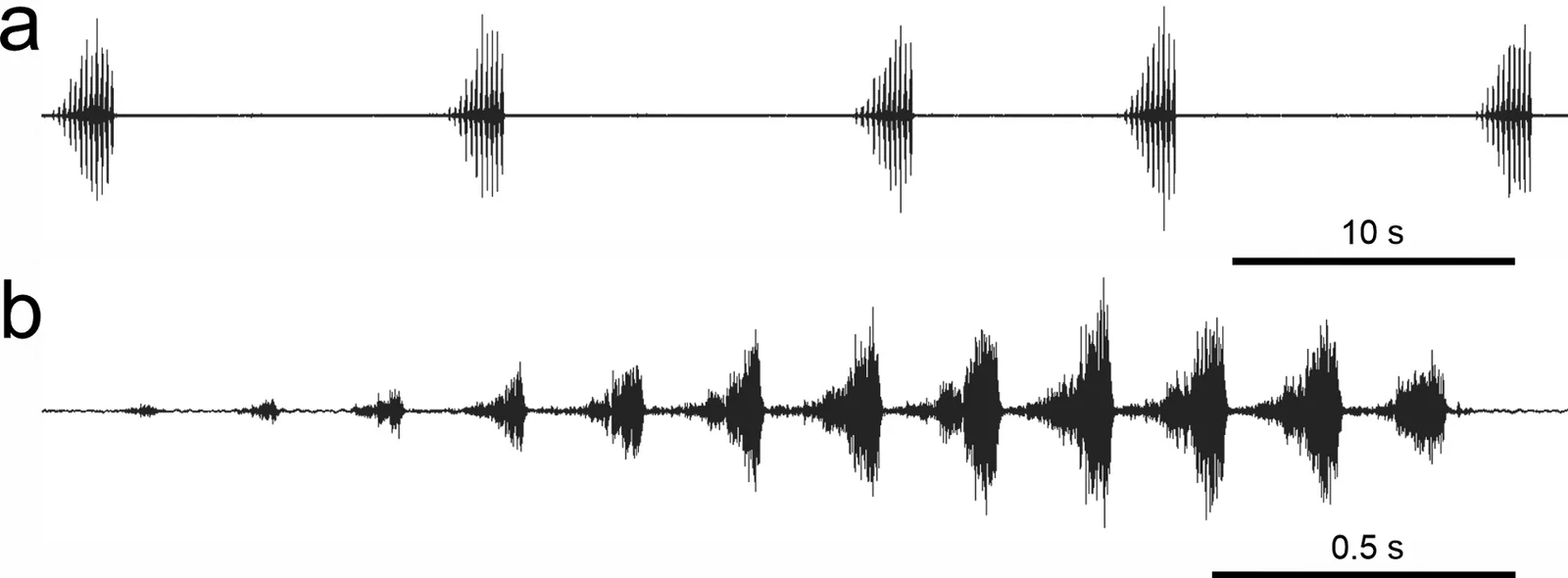
Oscillograms of the calling song of *Podismopsis kristini* sp. nov. **a**. Sequence of echemes; b. Detail of a single echeme. Recorded at 20–21 °C.

##### Diagnosis.

*Podismopsis kristini* sp. nov. resembles the other European species *P. frontalis*, *P. keisti*, *P. relicta*, *P. styriaca*, and *P. transsylvanica*. *Podismopsis poppiusi*, which extends its distribution into eastern Europe, differs from all the above-mentioned species by a distinctly longer and more slender hind femur in females (at least 5× as long as its maximum width) and, in males, by the tegmen having a conspicuously narrow subcostal field, ~ 1/3 as wide as the costal field, and a longer pronotum prozona, which is at least 1.5× as long as the metazona. *P. frontalis*, in which only females are known, differs from females of all other European congeners by a wide vertex, which is 3× as wide as the width of frontal ridge between antennae. Males of *P. relicta* and *P. transsylvanica* differ from those of *P. kristini* sp. nov., *P. styriaca*, and *P. keisti* in antennal length; the antennae are conspicuously shorter in *P. relicta* and *P. transsylvanica*, being only 1.2–1.3× as long as the combined length of the head and pronotum (vs ~ 1.5× in the remaining species). Males of *P. kristini* sp. nov. differ from males of *P. styriaca* in the relative width of the tegmen costal and subcostal fields (Fig. 8). The costal field of *P. kristini* sp. nov. and *P. keisti* is 1.3–1.7× as broad as the subcostal field in the distal third of the tegmen, whereas in *P. styriaca* this ratio is 1.9–2.0. The tegmina of *P. kristini* sp. nov. are, in contrast to those of *P. styriaca* and *P. keisti*, typically truncated apically, with the truncated margin distinctly emarginated (Fig. 8). Based on the examined material, we observed differences in the presence and number of spurious veins in the radial field of the tegmina among species. Although the formation of spurious veins shows considerable variability, certain arrangements predominate within each species. In *P. kristini* sp. nov. and *P. relicta* (Fig. 8a, c), the presence of two clearly distinguishable spurious veins in the radial field predominates, whereas in *P. transsylvanica* and *P. styriaca*, a single spurious vein positioned medially in the radial field is most common. In *P. keisti*, the greatest variability was observed: in some individuals two spurious veins are present, in others one, and in a large proportion of individuals no spurious vein is distinctly formed (Fig. 8d). Males of the European *Podismopsis* species differ in tegmina length and pronotum length (Fig. 4). Pronotum length along the median line is ≥ 3.1 mm in all examined specimens of *P. styriaca* and *P. transsylvanica*, whereas it is < 3.1 mm in all examined specimens of *P. kristini* sp. nov. Tegmina length is ≤ 8.6 mm in all measured specimens of *P. kristini* sp. nov. and > 8.6 mm in all measured specimens of *P. keisti* (Fig. 4). The genitalia of the European alpine *Podismopsis* species are very similar to one another. As their soft parts can be easily deformed during processing, it is important to examine the individual parts of the epiphallus from various angles when interpreting them. The phallic complex of *P. relicta* (Fig. 10c) differs from that of other European alpine species in having shorter, wider ancorae and short, robust lophi. *P. kristini* sp. nov. (Fig. 10a) shares with *P. styriaca* and *P. transsylvanica* a wide central lophus portion with a distinctly concave proximal part. This feature differs from the condition in *P. keisti* (Fig. 10d), which has a narrower, flatter central lophus portion. The proximal lophus projection is wider and more robust in *P. styriaca* (Fig. 10e) and *P. transsylvanica* than in *P. kristini* sp. nov. (Fig. 10a). Although the apical valves are variable in shape, which may also vary depending on the angle of view, two visually distinct types can be distinguished within the European alpine *Podismopsis* species. *P. keisti* and *P. kristini* sp. nov. (Fig. 10f) have apical valves that are very thin, apically narrowed, and more widely spaced apart in the middle. The other species, *P. transsylvanica*, *P. relicta*, and *P. styriaca* (Fig. 10g), have the apical valves more robust, less widely spaced, and less sharply pointed. In females, the tegmina of *P. kristini* sp. nov. extend distally into a conspicuous triangular projection, the ventral surface of which is deeply concave (Fig. 9a–d), whereas in the remaining European species, the distal part of the tegmina is shaped differently (Fig. 9e–h). The ventral margin of the tegmen of *P. kristini* sp. nov. forms an angle of 39–42° with the radial vein, similarly to *P. keisti*. In contrast, this angle is smaller (~ 35°) in *P. transsylvanica* and *P. relicta*, and considerably larger (~ 60°) in *P. styriaca*. The species can be distinguished by a combination of the characters listed above.

The calling song of *Podismopsis kristini* sp. nov. does not differ substantially from those of other European congeners in its basic characteristics, including echeme duration, number of syllables per echeme, and the gradual increase in sound intensity within an echeme (see also Discussion).

##### Description.

**Male** (Figs 1a, 2, 3a–d). Body of small size for genus *Podismopsis*, above olive green with black lateral stripe from eyes to apex of abdomen, below olive green to yellow green (Fig. 1a). Antennae unicoloured, brown with slightly blackish apex. Lateral carina of pronotum with pale margin; paranota blackish in dorsal two-thirds and olive green in ventral third. Tegmina unicolour, pale brown. Hind femora yellowish brown, lower side of femora yellow, upper side olive green; knees black; hind tibiae yellow with blackish base; spines yellow with black apex. Metric characters summarised in Table 1.

**Table 1.** Measurements of males and females of *Podismopsis kristini* sp. nov. from the type locality in Žiar Valley, the Western Tatras in Slovakia. Metric values are in mm.

**Measured parameter**	**Male (*n* = 12)**		**Female (*n* = 10)**	
	**Mean**	**Min–max**	**Mean**	**Min–max**
Total body length	14.98	14.08–16.19	18.29	15.51–19.52
Head length dorsally in middle	2.41	2.28–2.58	2.72	2.45–2.97
Width between eyes dorsally	1.10	1.01–1.19	1.58	1.32–1.77
Pronotum length in middle line	2.80	2.53–3.04	3.87	3.27–4.15
Pronotum width proximally	1.67	1.59–1.79	2.36	2.02–2.63
Pronotum width distally	2.15	1.97–2.38	3.45	3.02–3.78
Pronotum width at narrowest point dorsally	1.41	1.31–1.51	2.14	1.85–2.45
Left tegmen length laterally	8.07	7.12–8.60	4.37	3.67–5.08
Ovipositor length dorsally	–	–	1.21	0.97–1.41
Ovipositor length laterally	–	–	2.15	1.97–2.29
Vertical eye diameter laterally	1.28	1.23–1.34	1.39	1.25–1.53
Genae distance between eye and mandible	1.98	1.86–2.14	2.77	2.69–2.96
Femur length laterally	9.58	8.94–10.12	11.19	9.79–11.80
Tibia length laterally	8.25	7.59–8.85	9.48	9.00–10.04
Mesosternal interspace width	0.82	0.73–0.89	1.43	1.33–1.57
Mesosternal interspace length	0.49	0.44–0.54	0.78	0.75–0.82
Femur maximum width	2.23	2.10–2.32	2.57	2.17–2.77
Femur minimum width	0.93	0.87–1.02	1.10	0.95–1.18
Number of stridulatory pegs	116	87–136	–	–

Head (Fig. 2a–c) short; face in profile oblique. Frontal costa converging upward, narrow, the vertex is 1.7× as wide as the basal part of the frontal costa, and the vertex between eyes is 3.3× as wide as the upper part of frontal costa (Fig. 2c). Median ocellus below line connecting lower edges of eyes. Depression of vertex with distinct median carinula. Foveolae absent. Eyes oval, slightly longer than wide (Fig. 2a–c); vertical diameter of eye 0.5–0.8× as long as subocular furrow; interocular space 1.4–1.5× as wide as maximal width of eye in dorsal view. Antennae filiform, 19–21-segmented, reaching base of hind femora; mid segments of antennae 1.7–2.1× as long as wide.

Pronotum (Fig. 2a, b) crossed by main transverse furrow; prozona 1.3–1.4× as long as metazona; anterior margin of prozona rounded; posterior margin of metazona weakly excised near median carina; lateral carinae in prozona slightly and regularly concave; lateral carinae in metazona slightly convex; widest part of pronotum 1.5× as wide as narrowest part (Fig. 2b). Lateral lobes of pronotum 1.2–1.3× as long as high. Prosternal process absent. Mesosternal lobes 1.2–1.4× as wide as mesosternal interspace; mesosternal interspace transversal, 1.5–1.7× as wide as long; base of mesosternal interspace 1.5–1.6× as wide as width of metasternal interspace. Metasternal interspace square (Fig. 2e). Tegmina not reaching apex of abdomen, 2.7–2.9× as long as pronotum and 2.6–2.8× as long as wide; width of costal field 1.3–1.7× as wide as subcostal field in distal third of tegmen. Hind wings vestigial, narrow, 0.7–2.0 mm long. Macropterous form unknown. Hind femora 4.3× (4.1–4.5) as long as maximal width; inner lower keel with 87–136 stridulatory pegs; apex of knee lobe circular. Hind tibiae with 10 or 11 outer and 10 inner dorsal spines; apically two larger hooked spines on outer and inner margin. First tarsal segment as long as combined length of second and third segments (Fig. 2f). Arolium broad, reaching two-thirds of length of claws. Tympanum large, oval (Fig. 2d). Tenth abdominal tergite roundly incised medially, without furculae. Supraanal plate triangular with deep median longitudinal sulcus. Cerci conical, 2.8–3.3× as long as wide near base (Fig. 2h, i); subgenital plate as long as wide in ventral view (Fig. 2j).

Male genitalia (Fig. 3a–c). Epiphallus bridge-shaped; bridge narrow, arch-like; ancorae conical, almost reaching anterior projections; posterior projections wide, triangular; lophi large, kidney-shaped, narrowed in apical third and shallowly incised along inner margin. Cingulum sclerotised, consisting of broad arched apodemes, zygoma, and rami; zygoma broadly rounded apically. Valves of cingulum narrow, divided, apically pointed. Apical valves of penis narrow, with pointed apex curved inward; apical valves as long as cingular valves; basal and apical penis valves connected by curved, unbroken flexure.

**Female** (Figs 1b–e, 3e–i). Larger than male; colouration more variable. Body dorsally dark brown, grey-brown, or bronze-brown, ventrally olive green to yellowish; laterally with black or blackish lateral stripe from vertex to apex of abdomen; lateral colouration sometimes absent and body unicolour. Antennae brown with blackish apex. Lateral carina of pronotum with pale margin; paranota blackish in dorsal two-thirds. Tegmina grey-brown or bronze-brown. Hind femora in body colour; lower side of femora yellow; inner side with longitudinal black stripe near base; knees blackish brown. Hind tibiae yellow-orange; spines yellow with black apex. Ovipositor brown. Erythrism also recorded (Fig. 1e). Metric characters summarised in Table 1.

Head as in male but vertex between eyes 1.5× as wide as basal part of frontal costa and 3.5× as wide as its narrowest upper part; eyes oval, slightly longer than wide; interocular space 2.0–2.2× as wide as maximal width of eye in dorsal view (Fig. 3e). Antennae filiform, 21–22-segmented; mid segments of antennae 1.3–1.5× as long as wide. Prozona 1.3–1.4× as long as metazona; anterior margin of prozona rounded; posterior margin of metazona distinctly excised near median carina (Fig. 3e). Lateral lobes of pronotum 1.1–1.2× as long as high; lower side of lobes broadly rounded. Mesosternal lobes 1.1–1.2× as wide as mesosternal interspace. Mesosternal interspace transversal, 1.8–1.9× as wide as long. Metasternal interspace broad, 2.0–2.2× as wide as long. Tegmina about as long as or slightly longer than pronotum (0.95–1.28×), scale-like, 1.8–2.0× as long as wide, with pointed, elongated apex (Fig. 3f); hind wings absent. Hind femora 4.2–4.5× as long as maximal width. Hind tibiae with 11 or 12 outer and inner dorsal spines. Arolium nearly as long as claws. Tympanum large, oval. Supraanal plate triangular with wide median longitudinal sulcus (Fig. 3g, h). Cerci conical, 1.9–2.0× as long as wide near base. Subgenital plate elongated (Fig. 3i), with distinctly triangular posterior margin extending into long, needle-like protrusion continuing between bases of basivalvular plates; subgenital plate 1.5–1.8× as long as wide (measured without needle-like protrusion). Basivalvular plates 3.4× as long as wide. Ovipositor almost conical; valves divergent, curved in distal third; upper margin of dorsal valves and lower margin of ventral valves finely serrated (Fig. 3g–i).

##### Distribution.

Slovakia: Western Tatras Mts., upper part of Žiar Valley, vicinity of Žiarske pleso (lake) at 1,820–1,920 m a.s.l. Known only from the type locality.

##### Biology.

*P. kristini* sp. nov. inhabits wind-exposed alpine grasslands above the upper limit of the continuous *Pinus mugo* Turra, 1764 zone, predominantly on south-facing slopes. The vegetation is dense and low-statured, dominated by graminoids and ericaceous dwarf shrubs, which are particularly abundant around rock outcrops, exposed boulders, and small scree fields (Fig. 5). *Pinus mugo* occurs only sporadically as isolated shrubs or small patches at the upper limit of its distribution. Individuals of *P. kristini* sp. nov. are typically associated with the grassy sward. The orthopteran community at the study site comprised the following species: *Roeseliana roeselii* (Hagenbach, 1822), *Euthystira brachyptera* (Ocskay, 1826) and *Omocestus viridulus* (Linnaeus, 1758).

##### Stridulation.

The calling song of *Podismopsis kristini* sp. nov. (Fig. 6) consists of short echemes repeated at a rate of ~ 3–7 per minute with inter-echeme intervals of 6.5–16.0 s. Each echeme lasts 1.8–2.3 s (mean 2.0), has a relatively stable temporal structure, and consists of short pulses grouped into 9–13 syllables. At the beginning of each echeme, the sound is soft; it then reaches its maximum amplitude in the final two-thirds of the echeme duration, before decreasing slightly and terminating abruptly. The syllable duration and the syllable rate vary in the ranges of 0.17–0.18 s and 5.2–5.6 s^-1^, respectively.

##### Etymology.

This new species is dedicated to our friend, Anton Krištín, a leading orthopterologist from Zvolen, Slovakia. The specific epithet is a noun in the genitive case.

### Molecular differentiation and phylogenetic position of *Podismopsis kristini* sp. nov.

The COI sequences obtained from one male and one female paratype were deposited in GenBank under accession numbers PZ562680 and PZ562681, respectively.

For comparative analyses of pairwise genetic distances, COI sequences of *Podismopsis* species available in GenBank and the Barcode of Life Data System (BOLD) were included (Table 2). The dataset comprised sequences of *P. poppiusi*, *P. keisti*, *P. altaica*, and *P. genicularibus*, together with a representative of the closely related genus *Chrysochraon*. Pairwise *p*-distances for the COI barcode region are presented in Table 2. *Podismopsis kristini* sp. nov. showed the lowest genetic distance from *P. keisti* (0.7%). Its genetic distances from *P. poppiusi* and *P. altaica* were 1.5% and 1.7%, respectively. The East Asian *Chrysochraon dispar* and *P. genicularibus* differed from *P. kristini* sp. nov. by 6.4% and 6.6–6.8%, respectively.

**Table 2.** Pairwise sequence divergence (*p*-distance) of *Podismopsis* species for the examined part of the mitochondrial marker COI (543 bp). Sources of sequences: (GB) – GenBank, (BO) – BOLD.

**No**.	**Species / GenBank or BOLD accession no**.	**1**	**2**	**3**	**4**	**5**	**6**	**7**
1	*Podismopsis kristini* sp. nov. / PZ562680 (GB)	x						
2	*Podismopsis kristini* sp. nov. / PZ562681 (GB)	0.000	x					
3	*Podismopsis poppiusi* / AY738344 (GB)	0.015	0.015	x				
4	*Podismopsis keisti* / GBORT796-15 (BO)	0.007	0.007	0.018	x			
5	*Podismopsis altaica* / AY738343 (GB)	0.017	0.017	0.020	0.020	x		
6	*Chrysochraon dispar* / AY738345 (GB)	0.064	0.064	0.066	0.066	0.055	x	
7	*Podismopsis genicularibus* / KC261391 (GB)	0.066	0.066	0.064	0.068	0.057	0.051	x
8	*Podismopsis genicularibus* / KX272725 (GB)	0.068	0.068	0.066	0.070	0.059	0.050	0.002

**Figure 7.**
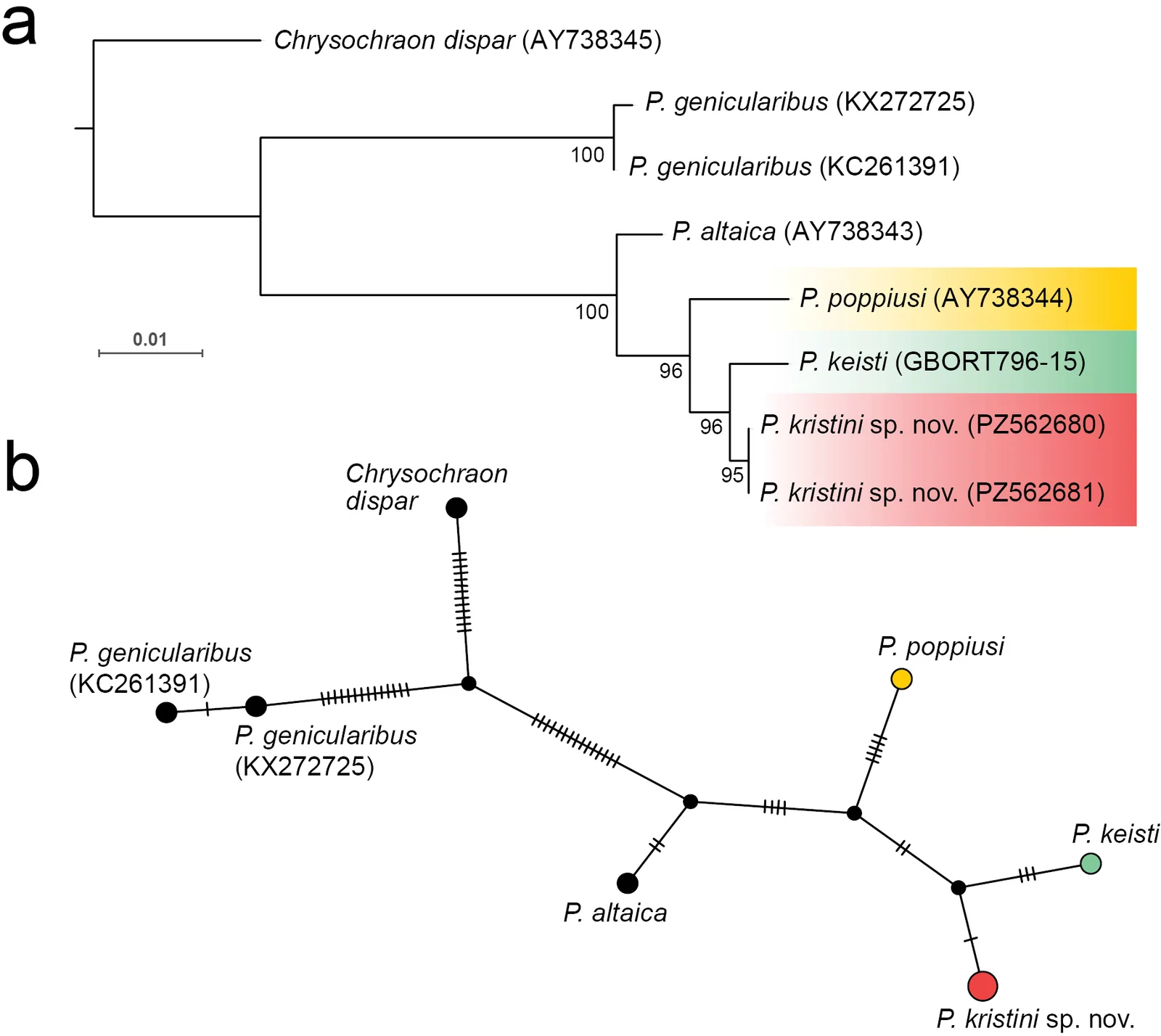
**a**. Maximum-likelihood phylogenetic tree inferred from the COI molecular marker. The alphanumeric code following each species name corresponds to the respective GenBank or BOLD accession number (see Table 2); **b**. Haplotype network based on the COI marker, illustrating interspecific relationships within *Podismopsis*. Hatch marks on the connections between haplotypes represent nucleotide substitutions. Each circle represents a unique haplotype; all haplotypes are represented by a single sequence, except for *P. kristini* sp. nov., which is represented by two identical sequences sharing the same haplotype.

The maximum-likelihood COI phylogeny recovered *Podismopsis kristini* sp. nov. as a distinct lineage, with its two sequences forming a well-supported clade (BS = 95). This clade was sister to *P. keisti* (BS = 96) and together formed a lineage sister to *P. poppiusi* (BS = 96); *P. altaica* was placed as sister to this group. The two *P. genicularibus* sequences formed a separate, strongly supported lineage (BS = 100). The COI haplotype network was congruent with the phylogeny, resolving each taxon as a distinct haplotype; *P. kristini* sp. nov. was closest to *P. keisti*, differing by four mutational steps (Fig. 7).

**Figure 8.**
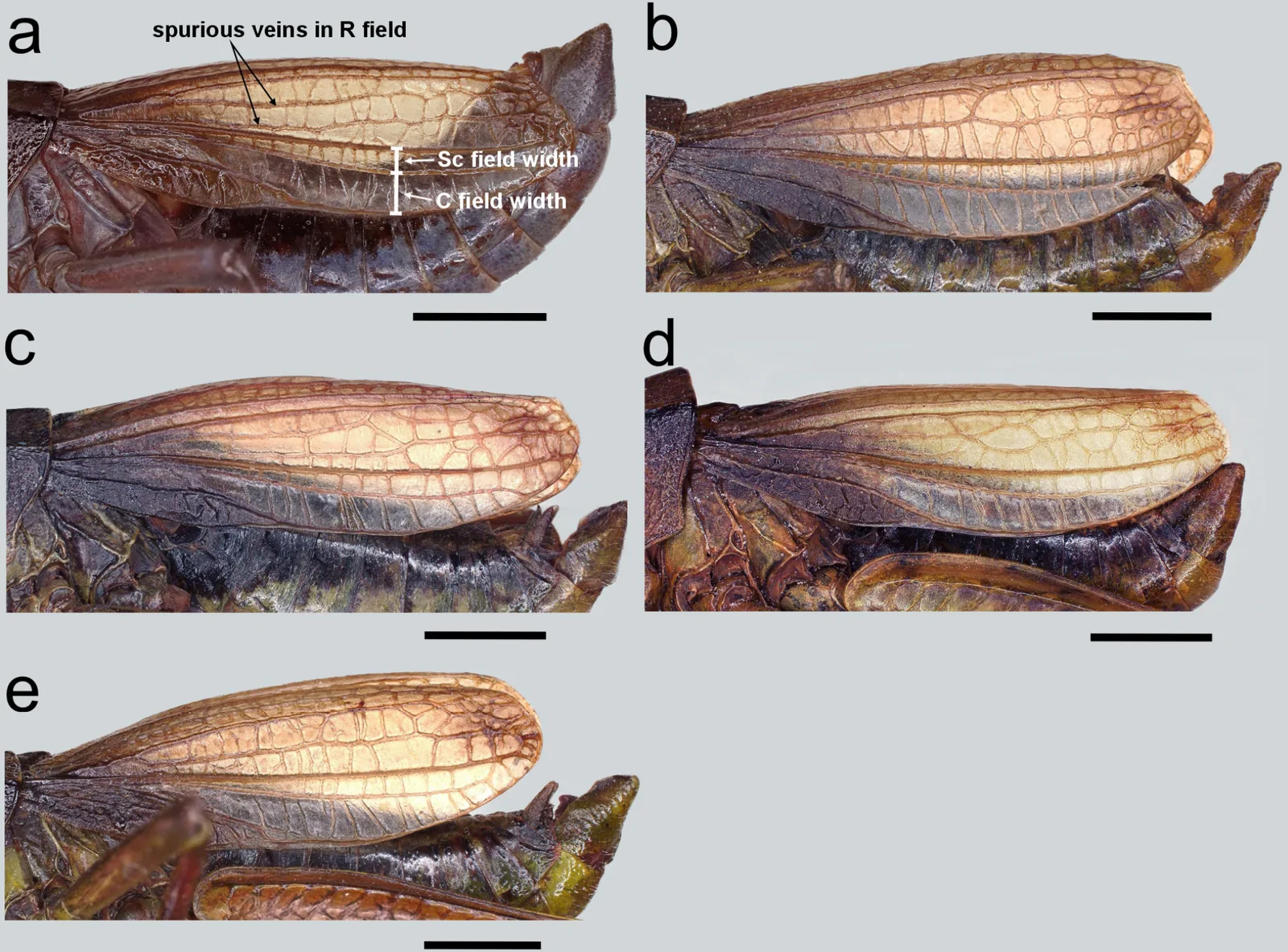
Lateral views of the left tegmen in males of *Podismopsis* spp. **a**. *Podismopsis kristini* sp. nov.; **b**. *P. transsylvanica* Ramme, 1951; **c**. *P. relicta* Ramme, 1931; **d**. *P. keisti* (Nadig, 1989); **e**. *P. styriaca* Koschuh, 2008. C – costal field, Sc – subcostal field, R – radial field. Scale bars: 2.0 mm.

**Figure 9.**
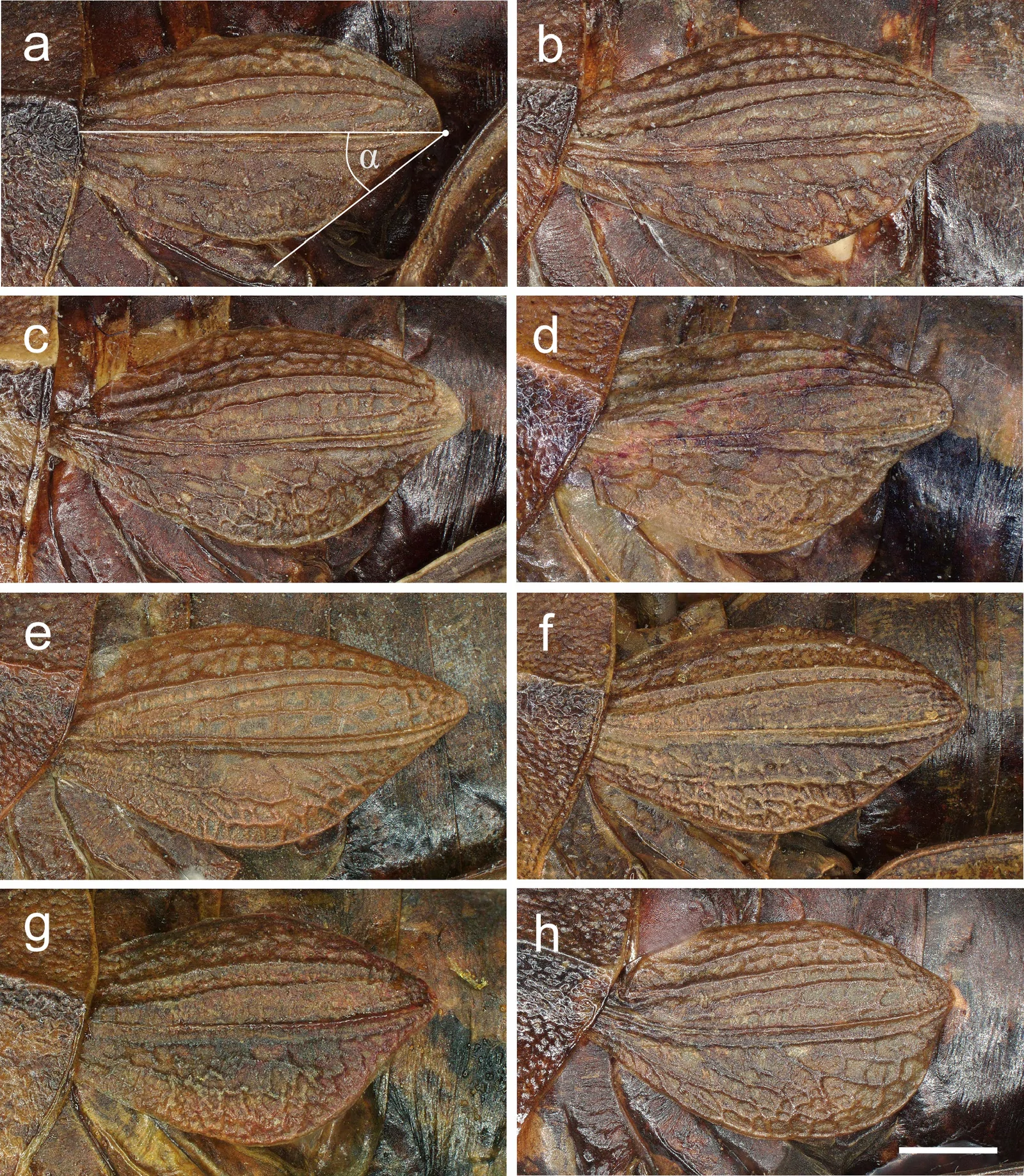
Lateral views of the left tegmen in females of *Podismopsis* spp. **a–d**. *Podismopsis kristini* sp. nov.; **e**. *P. transsylvanica* Ramme, 1951; **f**. *P. relicta* Ramme, 1931; **g**. *P. keisti* (Nadig, 1989); **h**. *P. styriaca* Koschuh, 2008. Abbreviation: α – the angle enclosed by the lower edge of the tegmen and the radial vein. Scale bar: 1.0 mm.

**Figure 10.**
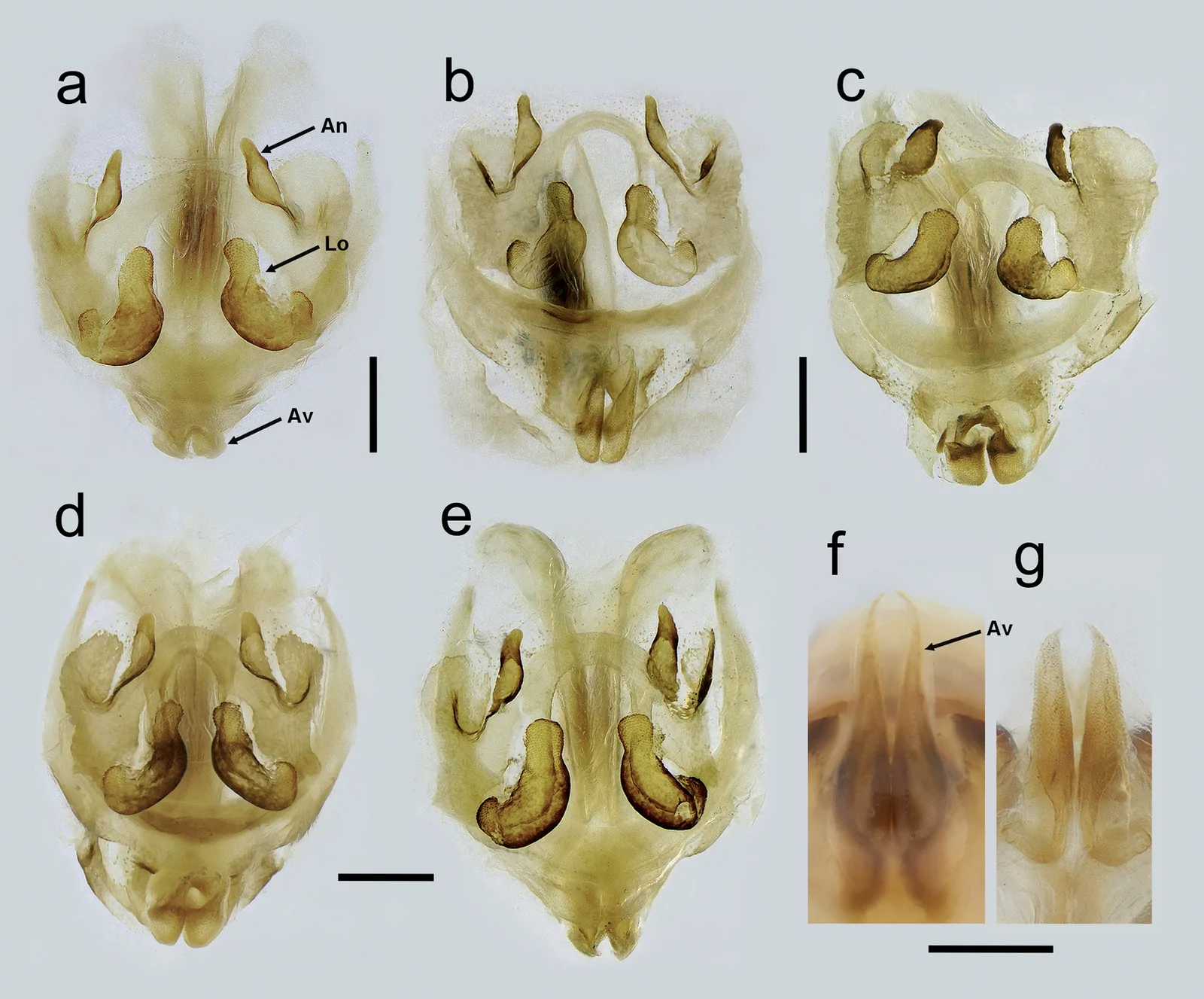
Male genitalia of *Podismopsis* spp. Dorsal views of the phallic complex of *Podismopsis* spp. (**a–е**): **a**. *Podismopsis kristini* sp. nov.; **b**. *P. transsylvanica* Ramme, 1951; **c**. *P. relicta* Ramme, 1931; **d**. *P. keisti* (Nadig, 1989); **e**. *P. styriaca* Koschuh, 2008; apical valves of penis in ventral view (**f, g**): **f**. *Podismopsis kristini* sp. nov.; **g**. *P. styriaca*. Abbreviations: An – ancora, Lo – lophus, Av – apical valve of penis. Scale bars: 0.5 mm.

### Identification key for European species of *Podismopsis*

**Table:** 

1	Male	**2**
–	Female	**7**
2	Tegmen with costal field about 3× as broad as subcostal field in distal third. Length of pronotum prozona at least 1.5× as long as metazona (along median carina). Distribution: Russia	***P. poppiusi* (Miram)**
–	Tegmen with costal field 1.3–2.5× as broad as subcostal field in distal third. Pronotum prozona 1.2–1.5× as long as metazona	**3**
3	Antennae shorter, 1.2–1.3× as long as head and pronotum combined; middle antennomeres as long as broad	**4**
–	Antennae long, ca 1.5× as long as head and pronotum combined; middle antennomeres distinctly longer than broad	**5**
4	Tegmen rounded apically or only slightly truncated (Fig. 8c); arolium small, reaching 1/2 length of claws. Distribution: Montenegro	***P. relicta* Ramme**
–	Tegmen truncated apically and slightly emarginated (Fig. 8b); arolium bigger, reaching 2/3 length of claws. Distribution: Romania	***P. transsylvanica* Ramme**
5	Tegmen costal field about 1.3–1.7× as broad as subcostal field in distal third of tegmen (Fig. 8a, d); apical valves of penis thin and sharply pointed (Fig. 10f)	**6**
–	Tegmen with costal field 1.9–2.0× as broad as subcostal field in distal third of tegmen (Fig. 8e); apical valves of penis more robust, less sharply pointed (Fig. 10g). Distribution: Austria	***P. styriaca* Koschuh**
6	Length of tegmen shorter than or equal to 8.6 mm. Tegmina truncated apically and distinctly emarginated at truncated edge (Fig. 3d). Distribution: Slovakia	***P. kristini* sp. nov**.
–	Tegmen longer than 8.6 mm. Tegmina rounded apically or slightly roundly truncated (Fig. 8d). Distribution: Switzerland	***P. keisti* (Nadig)**
7	Vertex 3× as wide as width of frontal ridge between antennae. Males unknown. Distribution: Russia	***P. frontalis* Mistshenko**
–	Vertex up to 2.8× as wide as width of frontal ridge between antennae	**8**
8	Hind femur ~ 5.0–5.5× as long as its maximal width. Distribution: Russia	***P. poppiusi* (Miram)**
–	Hind femur 4.2–4.6× as long as its maximal width	**9**
9	Tegmen distally extended into conspicuous triangular protrusion, ventral side of which is deeply concave (Fig. 9a–d). Lower edge of tegmen forms angle of 39–42° with radial vein. Distribution: Slovakia	***P. kristini* sp. nov**.
–	Tegmen roundly triangular distally, ventral edge straight or slightly concave (Fig. 9e–h)	**10**
10	Hind tibia yellowish	**11**
–	Hind tibia reddish or orange	**12**
11	Transverse furrow of pronotum straight or nearly straight. Distribution: Montenegro	***P. relicta* Ramme**
–	Transverse furrow of pronotum distinctly triangularly extended forward in middle. Distribution: Switzerland	***P. keisti* (Nadig)**
12	Tegmen as long as or shorter than pronotum. Distribution: Austria	***P. styriaca* Koschuh**
–	Tegmen longer than pronotum. Distribution: Romania	***P. transsylvanica* Ramme**

## Discussion

**Figure 11.**
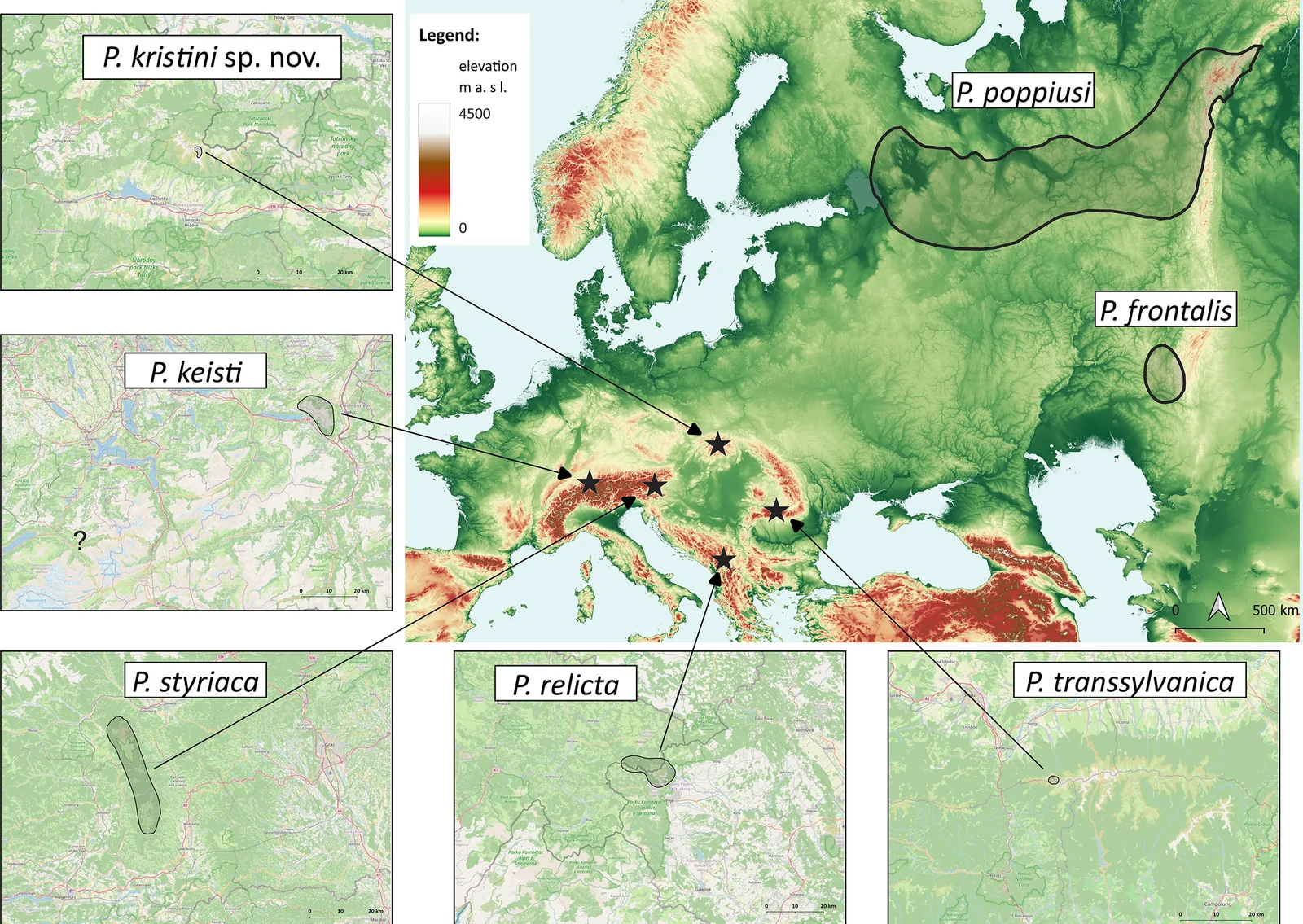
Distribution of European species of the genus *Podismopsis*. For *P. poppiusi*, only the European part of the distribution range is shown. The question mark denotes the location of a taxonomically unresolved population of *Podismopsis* sp. from Switzerland (Breitenmoser 2017). Basemap: Copernicus GLO-90 Digital Elevation Model (ESA 2021). Inset maps: OpenStreetMap (OpenStreetMap contributors 2026).

The seven European species of *Podismopsis* are distributed vicariantly across the Alps (Switzerland and Austria), the Western Carpathians (Slovakia), the Southern Carpathians (Romania), the Dinaric Alps (Montenegro), and the boreal Fennoscandian–East European region extending towards the Urals (northern Russia), see Fig. 11 (Ramme 1951; Bey-Bienko and Mistshenko 1951; Nadig 1989; Koschuh 2008; Gottsberger and Berger 2011). These species exhibit two principal distribution patterns.

*Podismopsis poppiusi* has a broad boreal Eurasian distribution, ranging from north-eastern Europe through western and eastern Siberia to the Altai–Sayan region. In Europe, the species is confined to northern European Russia, where its range extends from the Lake Onega–Vodlozersky National Park region eastwards across the northern East European Plain towards the Ural region (Hochkirch et al. 2016b). The isolated record from the Codrii Reserve in Moldova (Nicolaescu and Derjanschi 2001) is considered doubtful and has not been confirmed by subsequent surveys (Stahi and Derjanschi 2012). *Podismopsis poppiusi* is regarded as a common species with a stable population trend; however, local populations are generally small and highly fragmented (Hochkirch et al. 2016b). Throughout its range, the species inhabits forests and is typically associated with herbaceous vegetation in forest clearings (Hochkirch et al. 2016b).

In contrast, the Central and Southern European species have highly restricted distributions confined to montane and alpine habitats above 1,800 m a.s.l. in three European mountain systems (Fig. 11). *Podismopsis kristini* sp. nov. and *P. transsylvanica* inhabit the northern (the Tatras) and southern (the Făgăraș Mountains) margins of the Carpathian arc, respectively, with *P. kristini* sp. nov. representing the northernmost of the Central and Southern European species. The range of *P. relicta* is restricted to the Prokletije Mountains in the Dinaric Alps and represents the southernmost European species of the genus. Three additional *Podismopsis* taxa occur in the Alps. *Podismopsis styriaca* inhabits the southeastern margin of the Alps, whereas *P. keisti* occurs on the northwestern margin. Both species are associated with peripheral alpine mountain systems at elevations of ca 2,000 m a.s.l. and are absent from the inner high Alps. A further population of *Podismopsis* with unresolved taxonomic status occurs in Switzerland, ca 100 km southwest of the nearest known population of *P. keisti* (Breitenmoser 2017).

The status of *P. frontalis* remains particularly uncertain. The species was described in the mid-twentieth century based exclusively on female specimens collected from two localities in Bashkiria in the southern Ural Mountains at elevations between 280 and 600 m a.s.l. (Bey-Bienko and Mistshenko 1951). Its current distribution and population status remain unknown, although the species may be considerably more widespread than currently documented (Chobanov et al. 2016c).

The distribution ranges of the European species of *Podismopsis* do not overlap and are separated by considerable geographic distances. *Podismopsis kristini* sp. nov. is geographically isolated from all other known European congeners; the nearest congener is *P. styriaca*, whose distribution area lies ca 450 km away. The next closest congeners are *P. transsylvanica* and *P. relicta*, with minimum distances of ca 530 km and 700 km, respectively. The pairwise distances between all European *Podismopsis* taxa are summarised in Table 3.

**Table 3.** Geographic distances (in km) between the distribution areas of European *Podismopsis* species. The distances were measured between the closest edges of the areas. Geographic distances of *P. kristini* sp. nov. to European congeners are shaded.

	***Podismopsis frontalis***	***Podismopsis keisti***	***Podismopsis kristini***	***Podismopsis poppiusi***	***Podismopsis relicta***	***Podismopsis styriaca***	***Podismopsis transsylvanica***
*P. frontalis*	x						
*P. keisti*	3,250	x					
*P. kristini* sp. nov.	2,450	800	x				
*P. poppiusi*	800	2,100	1,500	x			
*P. relicta*	2,800	980	700	2,100	x		
*P. styriaca*	2,900	400	450	1,900	640	x	
*P. transsylvanica*	2,300	1,170	530	1,700	460	770	x
*Podismopsis* sp. (Switzerland)	3,350	100	900	2,200	1,050	480	1,260

The area of occupancy is very small in all narrowly distributed European species of *Podismopsis*. The largest values have been reported for *P. styriaca* (36 km^2^; Zuna-Kratky et al. 2016b) and *P. transsylvanica* (24 km^2^; Chobanov et al. 2016b), whereas *P. keisti* and *P. relicta* occupy only 10 km^2^ and 8 km^2^, respectively (Zuna-Kratky et al. 2016a; Chobanov et al. 2016a). *Podismopsis kristini* sp. nov. is currently known from an even smaller area, ca 1.5 km^2^.

The disjunct distribution pattern of *Podismopsis* spp. is characteristic of cold-adapted boreo-alpine species currently confined to isolated mountain summits, where they persist in Holocene refugia (Berger et al. 2010; Hagberg et al. 2022). Similar vicariant patterns have been documented in many other insects, for example, in the alpine leaf beetles of the genus *Oreina* Chevrolat in Dejean, 1837 (Chrysomelidae), whose lineages were divided during glacial refugia within and at the margins of the Alps and Apennines (Borer et al. 2010; Triponez et al. 2011), or in the mountain ringlet butterfly *Erebia epiphron* (Knoch, 1783) (Lepidoptera: Nymphalidae), which exhibits five genetically differentiated lineages corresponding to distinct Pleistocene refugial areas across the Pyrenees and Alps (Schmitt et al. 2006). The completely flightless earwigs of the genus *Chelidura* Latreille, 1825 (Dermaptera) provide a particularly instructive parallel: at least five morphologically and genetically distinct species are currently recognised in the Alps alone, each isolated within its own high-altitude range above 1,400–2,300 m a.s.l. (Fontana et al. 2026). Molecular and morphological analyses of the closely related genus *Chelidurella* Verhoeff, 1902 likewise reveal multiple cryptic species with restricted, allopatric distributions in the Alps, resulting from long-term geographic isolation driven by limited dispersal capacity in the absence of functional wings (Kirstová et al. 2021; Kočárek et al. 2026).

Among Orthoptera, the grasshoppers of the genus *Miramella* exhibit similar distributional patterns across European mountain systems, with a disjunct range spanning the Pyrenees, Alps, and Carpathians (Defaut and Morichon 2015). However, species of the genus *Miramella* do not show such a close association with specific microclimate conditions, and some individuals are macropterous, enabling better dispersal (Kral 2018). Consequently, populations are less isolated from each other, and in addition to mountain populations, several relict lowland populations occur in Europe (Reich 1991). Indeed, loss of flight has been shown to elevate speciation rates in insects significantly (Ikeda et al. 2012; Ortego and Knowles 2022), a pattern fully consistent with the evolutionary history of *Podismopsis*. The brachypterous condition shared by all *Podismopsis* species precludes dispersal to neighbouring mountain ranges, thereby restricting populations to patchily distributed alpine meadows (Gottsberger and Berger 2011).

The low levels of genetic and phenotypic differentiation observed among *Podismopsis* populations (Gottsberger and Berger 2011) suggest that their ancestor was broadly distributed across the glacial steppes extending between the northern ice sheet and the mountain glaciers to the south. This inference is directly corroborated by the subfossil record: the discovery of *Podismopsis pleistocenica* Zeuner, 1942 from Pleistocene woolly rhinoceros deposits at Starunia in the Carpathians (ca 37–40 ka BP; Zeuner 1934, 1942; Kuc et al. 2012) at only 404 m a.s.l. confirms the former lowland occurrence of the genus during the glacial periods (Nadig 1989). The current isolation of *Podismopsis* populations within discrete high-mountain systems is therefore a postglacial phenomenon, driven by rapid climatic warming at the Pleistocene–Holocene transition, which caused a dramatic upslope contraction of cold-adapted steppe communities (Hewitt 1999). Gene flow among the European mountain populations was thus interrupted as the species retreated into their respective high-altitude refugia following deglaciation, with the resulting allopatric isolation providing the conditions for the incipient divergence observed today (Gottsberger and Berger 2011). An analogous scenario has been proposed for Alpine *Chelidurella* earwigs, which are thought to have been derived from species currently distributed more widely outside the Alps (i.e., *C. acanthopygia* (Gené, 1832) and *C. thaleri* Harz, 1980): during glacial periods, populations were isolated in perialpine refugia, and following glacial retreat, the diverged lineages spread from these refugia, eventually becoming the isolated high-altitude endemics recognised today (Kirstová et al. 2021; Fontana et al. 2026). This scenario – widespread Pleistocene distribution followed by postglacial fragmentation – is consistent with the broader model of the “glacial species pump” documented for terrestrial endemics of the European Alps, where most insect endemics are of Pleistocene age and their current distributions reflect isolation in refugia following glacial retreat (Schmitt et al. 2006; Triponez et al. 2011; Kirstová et al. 2021).

Gottsberger and Berger (2011) reported low genetic distances among the species *Podismopsis keisti*, *P. relicta*, and *P. styriaca*, similar to our study, in which we found COI distances among *P. kristini* sp. nov., *P. keisti*, *P. poppiusi*, and *P. altaica* at the level of 0.7–2.0%. The lowest genetic distance of ~ 0.7% was found between *P. kristini* sp. nov. and *P. keisti*. For the *P. kristini*–*P. keisti* pair, such genetic distances are consistent with Pleistocene speciation and correspond to lineage splits ~ 200–300 thousand years ago according to Brower’s and Papadopoulou’s calibrations of molecular clocks for insect mtDNA (Brower 1994; Papadopoulou et al. 2010). These divergence estimates are consistent with the Pleistocene-driven speciation scenario proposed for *Italohippus* Fontana & La Greca, 1999 (Fontana and La Greca 1999; Forte et al. 2026), as well as with patterns documented in several other alpine and montane grasshopper radiations (e.g. Ortego et al. 2021). Morphological differences among these species are also minor and concern mainly morphometric traits that exhibit considerable intraspecific variability (Nadig 1989; Koschuh 2008; Gottsberger and Berger 2011). From a taxonomic perspective, such recently diverged taxonomic entities may be interpreted as species, subspecies, or as geographically isolated lineages of a single species, in which the speciation process is still ongoing (De Queiroz 2007; Joshi et al. 2024). In this paper, we follow the prevailing taxonomic concept that treats the isolated European populations as distinct species, as their large geographic separation, narrow association with specific microclimatic conditions, and limited dispersal capacity together provide sufficient reproductive isolation (Hewitt 2000; Dynesius and Jansson 2014; Ortego and Knowles 2022).

Low genetic distances between morphologically similar species are not unusual in Gomphocerinae and represent a consistent feature of lineages with Pleistocene histories. Gomphocerinae as a whole constitute an evolutionarily young radiation. Hawlitschek et al. (2022) estimated the age of the *Chorthippus biguttulus* (Linnaeus, 1758) complex at 1.38 Ma (95% HPD: 0.77–2.35 Ma), with the oldest divergence within the complex at 0.94 Ma, i.e., entirely within the Quaternary. Nolen et al. (2020) dated the divergence of *C. biguttulus*, *C. brunneus* (Thunberg, 1815), and *C. mollis* (Charpentier, 1825) to ~ 0.49–0.51 Ma and linked this radiation to climatic oscillations of the Middle Pleistocene. As a consequence of this young radiation, interspecific genetic distances in mitochondrial markers are very low. In an analysis of COI sequences in 50 Gomphocerinae species, Vedenina and Mugue (2011) found interspecific distances of 0.3–1.8% within the *C. biguttulus* group and 0.3–1.9% within the *C. albomarginatus* group; in some species pairs, interspecific divergence was lower than intraspecific variability within a single species. In the same vein, Sorokina et al. (2024) reported a mean COI*p*-distance within the genus *Stenobothrus* Fischer, 1853 of only 1.27 ± 0.25%, corresponding to a divergence of ~364 ka assuming a rate of 3.54% My^-1^ (Papadopoulou et al. 2010). Sharing of COI barcodes (BINs) among *C. biguttulus*, *C. brunneus*, *C. mollis*, *C. apricarius* (Linnaeus, 1758), and *Gomphocerippus rufus* (Linnaeus, 1758) was documented by Hawlitschek et al. (2017) and interpreted as a consequence of incomplete lineage sorting resulting from a very recent radiation. These examples from the tribes Gomphocerini and Stenobothrini collectively demonstrate that low COI distances (<1%) between morphologically and ecologically distinct species are a normal outcome of Pleistocene speciation in Gomphocerinae, rather than an indicator of conspecificity or inadequate taxonomic delimitation.

The calling song has been suggested to represent the most important component of reproductive isolation in Gomphocerinae grasshoppers (Vedenina and Mugue 2011). Within the genus *Podismopsis*, however, the basic characteristics of the calling song show little interspecific variation (Tishechkin 2008; Vedenina and Mugue 2011), suggesting that acoustic signals alone may not function as the primary isolating mechanism in this genus.

Koschuh (2008) considered the number of syllables per echeme to be an important diagnostic acoustic character distinguishing *Podismopsis styriaca* (4–5 syllables) from *P. keisti* (8–9 syllables) and *P. transsylvanica* (6–8 syllables). Published and publicly available recordings, however, indicate substantially broader variation in this character, with considerable overlap among species. Gottsberger and Berger (2011), based on recordings of *P. keisti*, *P. relicta*, and *P. styriaca*, reported that the number of syllables per echeme can range from a few to more than ten even within a single stridulating male, while the temporal pattern of the song also varies considerably both within and among *Podismopsis* populations. The number of syllables per echeme therefore does not appear to be a reliable diagnostic character for European *Podismopsis*. The calling song of *P. kristini* sp. nov. falls within this range of variation and shows no consistent diagnostic differences from those of its European congeners. Acoustic similarity extends beyond the European species: Tishechkin (2008) reported a high degree of similarity in the basic temporal pattern of calling songs among several Asian *Podismopsis* species, with considerable overlap in acoustic parameters, particularly between allopatric species.

Unlike the numerous cases of morphologically similar gomphocerine species distinguished by divergent acoustic signals, the reverse pattern–morphological differentiation accompanied by highly similar songs–appears to be less common in Acrididae (Şirin et al. 2014 and references therein). The acoustic conservatism observed among European *Podismopsis* may be related to their allopatric distributions, since selection for interspecific discrimination through calling songs is likely weak in geographically isolated lineages; morphological differentiation may therefore accumulate without a corresponding divergence in acoustic signals. A comparable pattern – pronounced morphological differentiation combined with a largely uniform calling song – has been documented in the allopatric bush-cricket genus *Eupholidoptera* Mařan, 1953 (Heller 2006; Çiplak et al. 2009; Willemse et al. 2023).

The pattern documented above – strictly allopatric, morphologically and ecologically differentiated mountain populations separated by only minimal mitochondrial divergence – corresponds closely to the concept of semi-species proposed by Lorković (1955). Lorković introduced this term for taxa that have reached an incomplete stage of speciation, i.e. an evolutionary condition intermediate between the subspecies (no reproductive isolation) and the species (complete reproductive isolation), in which reproductive isolation is only partial, and some gene exchange between the diverging populations would still be possible. He explicitly stressed that the criteria of the species concept can be applied unambiguously only to sympatric populations, whereas in allopatric populations the degree of reproductive isolation cannot be observed directly and any decision between “subspecies” and “species” remains inevitably arbitrary; the semi-species category was intended precisely for such borderline cases, and its scope, applicability and nomenclatural treatment were subsequently formalised by Lorković (1962). Significantly, Lorković (1955) developed the concept on a boreo-alpine model system closely comparable to ours, the *Erebia tyndarus* (Esper, 1781) complex (Lepidoptera, Nymphalidae), whose allopatric high-mountain lineages in the Pyrenees, Alps and Balkan Mountains differ only slightly in morphology, ecology and chorology while showing graded, incomplete reproductive isolation. The European species of *Podismopsis* fulfil this definition in all essential respects: they are completely allopatric and they differ consistently in a combination of morphometric and qualitative characters, yet show COI divergences of well below 2%; their calling songs remain essentially undifferentiated, so that no acoustic isolating mechanism has evolved; and their present isolation is purely geographical, maintained by brachyptery rather than by tested intrinsic barriers. They therefore represent a set of incipient species still in statu nascendi, i.e. semi-species collectively forming a superspecies in the sense of Mayr (1963) and Lorković (1955, 1962). Because semi-species and superspecies are not formally regulated ranks under the International Code of Zoological Nomenclature (ICZN 1999), taxa at this evolutionary stage are conventionally named at the species rank (Amadon 1966; Mallet 2021), the practice we follow here for *P. kristini* sp. nov.

## Supplementary Material

XML Treatment for Podismopsis

XML Treatment for Podismopsis kristini

## Additional information

### Artificial Intelligence (AI) use

The authors accept full responsibility for the content of the manuscript, including the disclosure of any use of AI.

Regarding the use of AI in the preparation of this manuscript, the authors declare the use of Perplexity for language, style and writing, literature discovery and brainstorming.

### Author ORCIDs

P. Kočárek https://orcid.org/0000-0002-1739-0143

P. Marhoul https://orcid.org/0009-0009-8909-8801

R. Vlk https://orcid.org/0009-0006-2861-1024

J. Holuša https://orcid.org/0000-0003-1459-0331

### Data availability

All of the data that support the findings of this study are available in the main text; the DNA sequences have been deposited in GenBank under accession numbers PZ562680–PZ562681.

## References

[B1] Amadon D (1966) The superspecies concept. Systematic Zoology 15(3): 245–249. 10.2307/2411398

[B2] Ander K (1949) Die boreo-alpinen Orthopteren Europas. Opuscula Entomologica 14: 89–104.

[B3] Baur B, Baur H, Roesti C, Roesti D (2006) Die Heuschrecken der Schweiz. Haupt, Bern, 352 pp.

[B4] Berger D, Chobanov DP, Mayer F (2010) Interglacial refugia and range shifts of the alpine grasshopper *Stenobothrus cotticus* (Orthoptera: Acrididae: Gomphocerinae). Organisms Diversity & Evolution 10: 123–133. 10.1007/s13127-010-0004-4

[B5] Bey-Bienko GY (1932) Orthoptera palaearctica critica XI. The group Chrysochraontes (Acrid.). Eos, Revista Española de Entomología 8: 43–92.

[B6] Bey-Bienko GY, Mistshenko LL (1951) Locusts and Grasshoppers of the U.S.S.R. and Adjacent Countries. Keys to the Fauna of the U.S.S.R. (Vols. 1, 2). Nauka, Moscow, 400 pp.

[B7] Borer M, Alvarez N, Buerki S, Margraf N, Rahier M, Naisbit RE (2010) The phylogeography of an alpine leaf beetle: Divergence within *Oreina elongata* spans several ice ages. Molecular Phylogenetics and Evolution 57(2): 703–709. 10.1016/j.ympev.2010.08.017 20807580

[B8] Breitenmoser S (2017) Découverte d’une nouvelle population de *Podismopsis* sp. dans les Alpes suisses (Bernois). Entomo Helvetica 10: 133–158. 10.5169/seals-986083

[B9] Brower AVZ (1994) Rapid morphological radiation and convergence among races of the butterfly *Heliconius erato* inferred from patterns of mitochondrial DNA evolution. Proceedings of the National Academy of Sciences USA 91(14): 6491–6495. 10.1073/pnas.91.14.6491 PMC44228 8022810

[B10] Chobanov DP, Hochkirch A, Iorgu IS, Ivković S, Kristin A, Lemonnier-Darcemont M, Pușkar T, Sirin D, Skejo J, Szövényi G, Vedenina V, Willemse LPM (2016a) *Podismopsis relicta*. The IUCN Red List of Threatened Species 2016: e.T16084527A70214414. 10.2305/IUCN.UK.2016-3.RLTS.T16084527A70214414.en [accessed 15 June 2026]

[B11] Chobanov DP, Hochkirch A, Iorgu IS, Ivković S, Kristin A, Lemonnier-Darcemont M, Pușkar T, Sirin D, Skejo J, Szövényi G, Vedenina V, Willemse LPM (2016b) *Podismopsis transsylvanica*. The IUCN Red List of Threatened Species 2016: e.T17818A13985210. 10.2305/IUCN.UK.2016-3.RLTS.T17818A13985210.en [accessed 15 June 2026]

[B12] Chobanov DP, Hochkirch A, Iorgu IS, Ivković S, Kristin A, Lemonnier-Darcemont M, Pușkar T, Sirin D, Skejo J, Szövényi G, Vedenina V, Willemse LPM (2016c) *Podismopsis frontalis*. The IUCN Red List of Threatened Species 2016: e.T68454064A70214334. 10.2305/IUCN.UK.2016-3.RLTS.T68454064A70214334.en [accessed 15 June 2026]

[B13] Cigliano MM, Braun H, Eades DC, Otte D (2026) Orthoptera Species File Online. Version 5.0/5.0. http://Orthoptera.SpeciesFile.org [accessed 15 June 2026]

[B14] Çiplak B, Heller K-G, Willemse F (2009) Review of the genus *Eupholidoptera* (Orthoptera, Tettigoniidae): Different genitalia, uniform song. Zootaxa 2156(1): 1–75. 10.11646/zootaxa.2156.1.1

[B15] Coray A, Thorens P (2001) Orthoptères de Suisse: Clé de Détermination. Fauna Helvetica 5, Neuchâtel, 236 pp.

[B16] De Queiroz K (2007) Species concepts and species delimitation. Systematic Biology 56(6): 879–886. 10.1080/10635150701701083 18027281

[B17] Defaut B, Morichon D (2015) Criquets de France (Orthoptera, Caelifera). Faune de France 97. Fédération Française des Sociétés de Sciences Naturelles, Paris, 740 pp.

[B18] Dynesius M, Jansson R (2014) Persistence of within-species lineages: a neglected control of speciation rates. Evolution 68(4): 923–934. 10.1111/evo.12316 24329123

[B19] European Space Agency [ESA] (2021) Copernicus GLO-90 Digital Elevation Model. European Space Agency. https://dataspace.copernicus.eu/ [accessed 15 June 2026]

[B20] Folmer O, Black M, Hoeh W, Lutz R, Vrijenhoek R (1994) DNA primers for amplification of mitochondrial cytochrome c oxidase subunit I from diverse metazoan invertebrates. Molecular Marine Biology and Biotechnology 3: 294–299. 7881515

[B21] Fontana P, La Greca M (1999) *Italohippus* n. gen. di Gomphocerinae dell’Appennino Centrale (Italia) (InsectaOrthopteraAcrididae). Atti dell’Accademia Roveretana degli Agiati, Classe di Scienze Matematiche, Fisiche e Naturali, serie B 9: 5–18.

[B22] Fontana P, Malagnini V, Pedrazzoli F, Kočárek P, Ruzzier E (2026) Current knowledge of the genus *Chelidura* Latreille, 1825 in the Alps, with descriptions of two new species (Dermaptera, Forficulidae). Bulletin of Insectology 79: 29–51. 10.3897/bull.insectology.182663

[B23] Forte F, Riccieri A, Trillo M, Freda F, Fontana P, Bologna MA, Ortego J (2026) Quaternary climate-driven diversification of alpine grasshoppers in the Central Apennines: insights into Mediterranean sky islands as centers of microendemism. Journal of Biogeography 53(5): e70250. 10.1111/jbi.70250

[B24] Garnier S, Alibert P, Audiot P, Prieur B, Rasplus J-Y (2004) Isolation by distance and sharp discontinuities in gene frequencies: implications for the phylogeography of an alpine insect species, *Carabus solieri*. Molecular Ecology 13(7): 1883–1897. 10.1111/j.1365-294X.2004.02212.x 15189211

[B25] Gottsberger B, Berger D (2011) Surviving at sky islands? Recent study questions ancient theories about the relict status of alpine grasshoppers in Europe (Acrididae, Gomphocerinae, *Podismopsis*). Poster presented at the 2011 ESA Annual Meeting, Reno, Nevada, November 13–16.

[B26] Hagberg L, Celemín E, Irisarri I, Hawlitschek O, Bella JL, Mott T, Pereira RJ (2022) Extensive introgression at late stages of species formation: Insights from grasshopper hybrid zones. Molecular Ecology 31: 2384–2399. 10.1111/mec.16406 35191134

[B27] Hawlitschek O, Morinère J, Lehmann GUC, Lehmann AW, Kropf M, Dunz A, Glaw F, Detcharoen M, Schmidt S, Hausmann A, Szucsich NU, Caetano-Wyler SA, Haszprunar G (2017) DNA barcoding of crickets, katydids and grasshoppers (Orthoptera) from Central Europe with focus on Austria, Germany and Switzerland. Molecular Ecology Resources 17(5): 1037–1053. 10.1111/1755-0998.12638 27863033

[B28] Hawlitschek O, Ortiz EM, Noori S, Webster KC, Husemann M, Pereira RJ (2022) Transcriptomic data reveals nuclear-mitochondrial discordance in Gomphocerinae grasshoppers (Insecta: Orthoptera: Acrididae). Molecular Phylogenetics and Evolution 170: e107439. 10.1016/j.ympev.2022.107439 35189365

[B29] Heller K-G (2006) Song evolution and speciation in bushcrickets. In: Drosopoulos S, Claridge MF (Eds) Insect Sounds and Communication: Physiology, Behaviour, Ecology, and Evolution. CRC Press, Boca Raton, 137–151.

[B30] Hewitt G (2000) The genetic legacy of the Quaternary ice ages. Nature 405(6789): 907–913. 10.1038/35016000 10879524

[B31] Hewitt GM (1999) Post-glacial re-colonization of European biota. Biological Journal of the Linnean Society 68(1–2): 87–112. 10.1111/j.1095-8312.1999.tb01160.x

[B32] Hochkirch A, Nieto A, García Criado M, Cálix M, Braud Y, Buzzetti FM, Chobanov D, Odé B, Presa Asensio JJ, Willemse L, Zuna-Kratky T, Barranco Vega P, Bushell M, Clemente ME, Correas JR, Dusoulier F, Ferreira S, Fontana P, García MD, Heller KG, Iorgu IȘ, Ivković S, Kati V, Kleukers R, Krištín A, Lemonnier-Darcemont M, Lemos P, Massa B, Monnerat C, Papapavlou KP, Prunier F, Pushkar T, Roesti C, Rutschmann F, Şirin D, Skejo J, Szövényi G, Tzirkalli E, Vedenina V, Barat Domenech J, Barros F, Cordero Tapia PJ, Defaut B, Fartmann T, Gomboc S, Gutiérrez-Rodríguez J, Holuša J, Illich I, Karjalainen S, Kočárek P, Korsunovskaya O, Liana A, López H, Morin D, Olmo-Vidal JM, Puskás G, Savitsky V, Stalling T, Tumbrinck J (2016a) European Red List of Grasshoppers, Crickets and Bush-crickets. Publications Office of the European Union, Luxembourg. 10.2779/60944

[B33] Hochkirch A, Vedenina V, Pușkar T, Willemse LPM, Sirin D, Ivković S, Lemonnier-Darcemont M, Chobanov DP, Iorgu IS, Kristin A, Skejo J (2016b) *Podismopsis poppiusi* (Europe assessment). The IUCN Red List of Threatened Species 2016: e.T16084512A74509848 [accessed 15 June 2026].

[B34] Holdhaus K (1912) Kritisches Verzeichnis der boreoalpinen Tierformen (Glazialrelikte) der mittel- und südeuropäischen Hochgebirge. Annalen des Kaiserlich-Königlichen Hofmuseums 26: 399–440.

[B35] ICZN (1999) International Code of Zoological Nomenclature (4^th^ ed.). International Trust for Zoological Nomenclature, Natural History Museum, London, 306 pp. 10.5962/bhl.title.50608

[B36] Ikeda H, Nishikawa M, Sota T (2012) Loss of flight promotes beetle diversification. Nature Communications 3(1): 648. 10.1038/ncomms1659 PMC3272566 22337126

[B37] Joshi M, Espeland M, Huemer P, deWaard J, Mutanen M (2024) Species delimitation under allopatry: Genomic insights within and across continents in Lepidoptera. Insect Systematics and Diversity 8(5): 1–7. 10.1093/isd/ixae027

[B38] Kirstová M, Kundrata R, Kočárek P (2021) Molecular phylogeny and classification of *Chelidurella* Verhoeff, stat. restit. (Dermaptera: Forficulidae). Insect Systematics & Evolution 52(3): 335–371. 10.1163/1876312X-bja10004

[B39] Kočárek P, Fontana P, Kočárková I, Bonczek V (2026) A new cryptic species of *Chelidurella* Verhoeff, 1902 (Dermaptera, Forficulidae) from the Italian Alps: Molecular evidence reveals hidden diversity in a high-altitude refugium. ZooKeys 1272: 173–188. 10.3897/zookeys.1272.180451 PMC12988427 41837206

[B40] Koschuh A (2008) *Podismopsis styriaca* nov. sp. (Orthoptera, Acridinae) – ein Endemit im Ostalpenraum. Linzer Biologische Beiträge 40(1): 627–638. 10.48580/d78s

[B41] Kral K (2018) Ecological requirements and features adapting the Karinthian Mountain Grasshopper *Miramella carinthiaca* to live in meadows at the alpine treeline. Acta Entomologica Slovenica 26(2): 205–218.

[B42] Kuc T, Różański K, Kotarba MJ, Goslar T, Kubiak H (2012) Radiocarbon dating of Pleistocene fauna and flora from Starunia, SW Ukraine. Radiocarbon 54(1): 123–136. 10.2458/azu_js_rc.v54i1.13084

[B43] Kumar S, Stecher G, Suleski M, Sanderford M, Sharma S, Tamura K (2024) MEGA12: Molecular Evolutionary Genetic Analysis. Version 12 for Adaptive and Green Computing. Molecular Biology and Evolution 41(12): msae263. 10.1093/molbev/msae263 PMC11683415 39708372

[B44] Leigh JW, Bryant D (2015) POPART: A full-feature software for haplotype network construction. Methods in Ecology and Evolution 6: 1110–1116. 10.1111/2041-210X.12410

[B45] Lorković Z (1955) Semispecies, a necessary new taxonomic category. Biološki Glasnik 7: 236–237.

[B46] Lorković Z (1962) The genetics and reproductive isolating mechanisms of the *Pieris napi-bryoniae* group. Journal of the Lepidopterists’ Society 16(1): 5–19. [16(2): 105–127.]

[B47] Lu Y, Wang L, Ren BZ (2011) Review and key of East Palaearctic species of the genus *Podismopsis* Zubovsky (Orthoptera: Acridoidea) with description of a new species from China. Entomologica Fennica 22(3): 199–208. 10.33338/ef.4697

[B48] Mallet J (2021) Subspecies, semispecies, superspecies. In: Scheiner SM (Ed.) Encyclopedia of Biodiversity (Vol. 7, 3^rd^ edn.). Elsevier, Oxford, 546–549.

[B49] Mayr E (1963) Animal Species and Evolution. Belknap Press of Harvard University Press, Cambridge, 797 pp. 10.4159/harvard.9780674865327

[B50] Miram E (1907) Zur Orthopteren-Fauna Russlands. Öfversikt af Finska Vetenskaps-Societetens Förhandlingar 49(6): 1–9.

[B51] Nadig A (1989) Eine aus den Alpen bisher unbekannte Untergattung in der Schweiz – Chrysochraon (Podismopsis) keisti sp. n. (Saltatoria, Acridinae). Mitteilungen der Schweizerischen Entomologischen Gesellschaft 62: 79–86.

[B52] Nicolaescu V, Derjanschi V (2001) Contribuții la cunoaşterea faunei şi ecologiei ortopterelor (Orthoptera) din rezervația Codrii. In: Diversitatea, valorificarea raţională şi protecţia lumii animale. CE USM, Chişinău, 146 pp.

[B53] Nolen ZJ, Yildirim B, Irisarri I, Liu S, Groot Crego C, Amby DB, Mayer F, Gilbert MTP, Pereira RJ (2020) Historical isolation facilitates species radiation by sexual selection: Insights from *Chorthippus* grasshoppers. Molecular Ecology 29(24): 4985–5002. 10.1111/mec.15695 33065760

[B54] OpenStreetMap contributors (2026) OpenStreetMap. https://www.openstreetmap.org [accessed 15 June 2026].

[B55] Ortego J, Knowles LL (2022) Geographical isolation versus dispersal: Relictual alpine grasshoppers support a model of interglacial diversification with limited hybridization. Molecular Ecology 31(1): 296–312. 10.1111/mec.16225 34651368

[B56] Ortego J, Gutiérrez-Rodríguez J, Noguerales V (2021) Demographic consequences of dispersal-related trait shift in two recently diverged taxa of montane grasshoppers. Evolution 75(8): 1998–2013. 10.1111/evo.14205 33646593

[B57] Papadopoulou A, Anastasiou I, Vogler AP (2010) Revisiting the insect mitochondrial molecular clock: the mid-Aegean trench calibration. Molecular Biology and Evolution 27(7): 1659–1672. 10.1093/molbev/msq051 20167609

[B58] Pucher GM (2014) Erstnachweis der Steirischen Goldschrecke (*Podismopsis styriaca* Koschuh, 2008) für Kärnten. Carinthia II 204(124): 585–588.

[B59] QGIS Development Team (2025) QGIS Geographic Information System. Open Source Geospatial Foundation Project. https://qgis.org

[B60] Ramme W (1931) Eine *Podismopsis* (Orth. Acrid.) aus Südeuropa. Zoologischer Anzeiger 93: 123–126.

[B61] Ramme W (1951) Zur Systematik, Faunistik und Biologie der Orthopteren von Südost-Europa und Vorderasien. Mitteilungen aus dem Zoologischen Museum in Berlin 27: 1–431. 10.1515/9783112730034

[B62] Reich M (1991) Grasshoppers (Orthoptera, Saltatoria) on alpine and dealpine riverbanks and their use as indicators for natural floodplain dynamics. Regulated Rivers: Research & Management 6(4): 333–339. 10.1002/rrr.3450060411

[B63] Schmitt T, Hewitt GM, Müller P (2006) Disjunct distributions during glacial and interglacial periods in mountain butterflies: *Erebia epiphron* as an example. Journal of Evolutionary Biology 19(1): 108–113. 10.1111/j.1420-9101.2005.00980.x 16405582

[B64] Şirin D, Mol A, Akyıldız G (2014) The morphological and behavioral analysis of geographically separated *Rammeihippus turcicus* (Orthoptera: Acrididae: Gomphocerinae) populations: data result in taxonomical conflict. Journal of Insect Science 14(145): 1–15. 10.1093/jisesa/ieu007 PMC5443474 25347836

[B65] Sorokina S, Sevastianov N, Tarasova T, Vedenina V (2024) The fast evolution of the Stenobothrini grasshoppers (Orthoptera, Acrididae, Gomphocerinae) revealed by an analysis of the control region of mtDNA, with an emphasis on the *Stenobothrus eurasius* group. Insects 15(8): 592. 10.3390/insects15080592 PMC11354746 39194797

[B66] Stahi N, Derjanschi V (2012) The spatial structure of orthopteran communities from the Scientific Reserve Codrii from the Republic of Moldova. Oltenia – Studii şi Comunicări Ştiinţele Naturii 28(2): 57–62.

[B67] Taberlet P, Fumagalli L, Wust-Saucy A-G, Cosson J-F (1998) Comparative phylogeography and postglacial colonization routes in Europe. Molecular Ecology 7(4): 453–464. 10.1046/j.1365-294x.1998.00289.x 9628000

[B68] Taylor EH (1942) “Island” faunas on the Mexican Plateau. Proceedings of the 8^th^ American Scientific Congress 3: 503–504.

[B69] Tishechkin DY (2008) Calling songs of grasshoppers of the genus *Podismopsis* (Orthoptera: Acrididae: Gomphocerinae) and potentialities of use of acoustic characters for discrimination between species of the genus. Russian Entomological Journal 17(3): 259–272.

[B70] Triponez Y, Buerki S, Borer M, Naisbit RE, Rahier M, Alvarez N (2011) Discordances between phylogenetic and morphological patterns in alpine leaf beetles attest to an intricate biogeographic history of lineages in postglacial Europe. Molecular Ecology 20(11): 2442–2463. 10.1111/j.1365-294X.2011.05096.x 21518061

[B71] Vedenina V, Mugue NS (2011) Speciation in gomphocerine grasshoppers: Molecular phylogeny versus bioacoustics and courtship behavior. Journal of Orthoptera Research 20(1): 109–125. 10.1665/034.020.0111

[B72] Willemse L, Tilmans J, Kotitsa N, Trichas A, Heller K-G, Chobanov D, Odé B (2023) A review of *Eupholidoptera* (Orthoptera, Tettigoniidae) from Crete, Gavdos, Gavdopoula, and Andikithira. ZooKeys 1151: 67–158. 10.3897/zookeys.1151.97514 PMC10207466 37235177

[B73] Zeuner FE (1934) Die Orthopteren aus der diluvialen Nashornschicht von Starunia (Polnische Karpathen). Starunia 3: 1–17.

[B74] Zeuner FE (1942) The fossil Acrididae (Orth. Salt.). Part III. Acridinae. Annals and Magazine of Natural History, Series 11, 9: 304–314. 10.1080/03745481.1942.9755472

[B75] Zuna-Kratky T, Fontana P, Roesti C, Braud Y, Hochkirch A, Monnerat C, Rutschmann F, Presa JJ (2016a) *Podismopsis keisti*. The IUCN Red List of Threatened Species 2016: e.T16084435A70646072. 10.2305/IUCN.UK.2016-3.RLTS.T16084435A70646072.en [accessed 15 June 2026]

[B76] Zuna-Kratky T, Fontana P, Roesti C, Braud Y, Hochkirch A, Monnerat C, Rutschmann F, Presa JJ (2016b) *Podismopsis styriaca*. The IUCN Red List of Threatened Species 2016: e.T16084476A70646131. 10.2305/IUCN.UK.2016-3.RLTS.T16084476A70646131.en [accessed 15 June 2026]

